# YALIcloneNHEJ: An Efficient Modular Cloning Toolkit for NHEJ Integration of Multigene Pathway and Terpenoid Production in *Yarrowia lipolytica*

**DOI:** 10.3389/fbioe.2021.816980

**Published:** 2022-03-02

**Authors:** Ya-Wen Li, Cai-Ling Yang, Qi Shen, Qian-Qian Peng, Qi Guo, Zhi-Kui Nie, Xiao-Man Sun, Tian-Qiong Shi, Xiao-Jun Ji, He Huang

**Affiliations:** ^1^ School of Food Science and Pharmaceutical Engineering, Nanjing Normal University, Nanjing, China; ^2^ College of Biotechnology and Pharmaceutical Engineering, Nanjing Tech University, Nanjing, China; ^3^ Jiangxi New Reyphon Biochemical Co., Ltd., Salt and Chemical Industry, Xingan, China; ^4^ College of Pharmaceutical Sciences, Nanjing Tech University, Nanjing, China

**Keywords:** *Y. lipolytica*, (-)-α-bisabolol, sesquiterpene, Golden Gate cloning, non-homologous end-joining

## Abstract

Non-homologous end-joining (NHEJ)-mediated random integration in *Yarrowia lipolytica* has been demonstrated to be an effective strategy for screening hyperproducer strains. However, there was no multigene assembly method applied for NHEJ integration, which made it challenging to construct and integrate metabolic pathways. In this study, a Golden Gate modular cloning system (YALIcloneNHEJ) was established to develop a robust DNA assembly platform in *Y. lipolytica*. By optimizing key factors, including the amounts of ligase and the reaction cycles, the assembly efficiency of 4, 7, and 10 fragments reached up to 90, 75, and 50%, respectively. This YALIcloneNHEJ system was subsequently applied for the overproduction of the sesquiterpene (-)-α-bisabolol by constructing a biosynthesis route and enhancing the flux in the mevalonate pathway. The resulting strain produced 4.4 g/L (-)-α-bisabolol, the highest titer reported in yeast to date. Our study expands the toolbox of metabolic engineering and is expected to enable a highly efficient production of various terpenoids.

**GRAPHICAL ABSTRACT:**
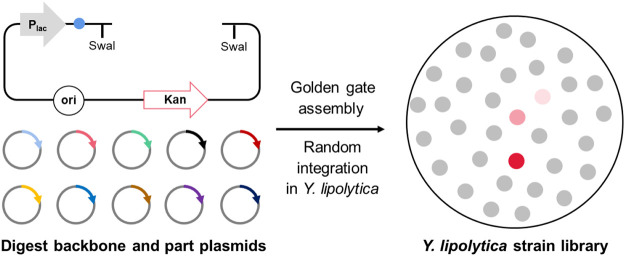


## Introduction

The monocyclic sesquiterpene (-)-α-bisabolol has important applications in the fields of medicine, food, and biofuels (Han et al., 2016; Lim et al., 2021)—for example, Melo et al. (2017) found that oral formulations of (-)-α-bisabolol can reduce oral injury in rodents to a certain extent. Tabari and Tehrani (2017) were the first to explore the potential mechanism of the pharmacological properties of (-)-α-bisabolol, showing that it could interact with the neurotransmitter γ-aminobutyric acid, exhibiting anxiolytic and sedative effects. In previous studies, (-)-α-bisabolol was mainly produced through plant extraction, but this method is limited due to low extraction efficiency and high cost (Lim et al., 2021). In recent years, with the fast development of synthetic biology, microbial fermentation has become increasingly more popular in the synthesis of natural products (Luo et al., 2015; Ledesma-Amaro and Nicaud, 2016; Chae et al., 2017). The synthesis of (-)-α-bisabolol has been previously achieved in *Escherichia coli* with the highest titer of 23.4 g/L (Lim et al., 2021). However, due to the possible infection risk of *E. coli* by bacteriophage, lots of researchers preferred to use fungi as the host to synthesize (-)-α-bisabolol—for example, some researchers attempted to use *Saccharomyces cerevisiae* to synthesize (-)-α-bisabolol heterologously. When a (-)-α-bisabolol synthase from *Matricaria recutita* was expressed, the titer was only 8 mg/L (Son et al., 2014). Introducing a truncated *HMG1* gene coding for HMG-CoA reductase, *ERG10* encoding acetyl-CoA thiolase, and *ACS1* gene coding for acetyl-CoA synthetase into *S. cerevisiae* genome, the titer increased to 124 mg/L (Kim et al., 2021). In order to improve the level, metabolic engineering strategies, including optimizing the mevalonate pathway and the lipid synthesis pathway, were adopted. The (-)-α-bisabolol titer was improved from 8 to 364 mg/L in yeast (Ma et al., 2021). Nevertheless, the production of (-)-α-bisabolol in fungi is still very low (Table 1).

**TABLE 1:** (-)-α-Bisabolol production in yeast.

Strains	Titer (mg/L)	Productivity (mg L^−1^ h^−1^)	References
*S. cerevisiae*	8	-	Son et al. (2014)
*S. cerevisiae* DtEMA	124	2.1	Kim et al. (2021)
*Y. lipolytica* Po1f-2BtHE-S-R8	364.23	3.04	Ma et al. (2021)
*Y. lipolytica* LYW7-3	4,400	26.20	This work

The non-conventional oleaginous yeast *Yarrowia lipolytica* is a generally-recognized-as-safe organism, and it has gradually become a promising host for the synthesis of various terpene chemicals (Ma et al., 2019; Li et al., 2021). Compared with *S. cerevisiae* and *Escherichia coli*, *Y. lipolytica* has many natural advantages, including a sufficient intracellular acetyl-CoA/cofactor supply, no phage infection risk, and no Crabtree effect (Cao et al., 2017; Tang et al., 2021). Therefore, *Y. lipolytica* has great potential as a robust chassis for biosynthesizing terpenes.

Although episomal plasmids containing centromeric sequences or autonomously replicating sequences have been developed for *Y. lipolytica*, they are usually unstable and have a low copy number (Dulermo et al., 2017). Consequently, the genetic manipulation of *Y. lipolytica* for metabolic engineering applications mainly relies on homologous recombination (HR)-mediated chromosomal integration (Larroude et al., 2018). However, this method is limited by low integration efficiency, especially in the case of long DNA fragments encoding multi-gene transcription units. Cui et al. (2019) systematically investigated a genomic integration method based on non-homologous end-joining (NHEJ) in *Y. lipolytica*. In comparison with homologous recombination, NHEJ has many unique advantages, including high integration efficiency and no need for a homologous template (Liu et al., 2019). Moreover, the heterologous DNA can be randomly inserted into the chromosome, which results in a library of multilocus integrants that can facilitate the screening of hyperproducer strains (Ye et al., 2012; Friedlander et al., 2016; Cui et al., 2021). However, there was no multigene plasmid construction method for NHEJ-based integration, which made it challenging to construct and integrate metabolic pathways in *Y. lipolytica*. Golden Gate is a gene cloning method using type IIS restriction endonucleases, which enables the modular assembly of customized DNA building blocks (Sarkari et al., 2017; Tong et al., 2021). By designing various features of the building block, including promoters with a highly variable strength of expression and terminators with a rigorously variable control of mRNA half-life, the Golden Gate assembly method can be applied into the combinatorial expression of multiple genes or the construction of gene libraries with variable intensities—for example, Celińska et al. (2017) designed a Golden Gate assembly system to create HR-mediated multigene integration for *Y. lipolytica*. Subsequently, the Golden Gate-based promoter shuffling was used to identify the best promoter set for the production of β-carotene (Larroude et al., 2017). The β-carotene titer could reach 6.5 g/L in *Y. lipolytica*, which is the highest yield currently reported in the literature. In addition, this assembly method also has the characteristics of low cost and convenient operation. Therefore, we aimed to establish a Golden Gate library specifically for NHEJ integration in *Y. lipolytica*.

In this work, we first adapted the Golden Gate method for NHEJ-based random integration in *Y. lipolytica*, which can be applied for the rapid assembly of up to 10 fragments. Using this approach, we rapidly optimized the biosynthetic pathway of (-)-α-bisabolol and realized a titer of 4.4 g/L, the highest (-)-α-bisabolol titer reported in yeast to date. This study expands the synthetic biology toolbox for DNA assembly and integration, greatly facilitating the genetic manipulation of the non-conventional yeast *Y. lipolytica*.

## Materials and Methods

### Strains, Media, and Culture Conditions

*E. coli* DH5α was used for plasmid construction. The strains with plasmids encoding gene fragments were cultivated at 37°C in lysogeny broth (LB) medium supplemented with 100 μg/ml ampicillin, while the strains with the backbone plasmids with or without assembled building blocks were cultivated at 37°C in LB supplemented with 50 μg/ml kanamycin, 40 μg/ml X-Gal, and 0.05 μM IPTG. The Po1h (uracil auxotrophy) and other *Y. lipolytica* strains were cultivated at 30°C with shaking at 240 rpm in YPD medium for seed culture (20 g/L peptone, 10 g/L yeast extract, and 20 g/L glucose). Subsequently, the seed cultures were transferred into the fermentation medium YPDE (20 g/L peptone, 10 g/L yeast extract, and 60 g/L glucose) at an initial OD_600_ of ∼0.1, followed by a fermentation period of 4 days. An upper organic phase comprising 10 ml dodecane was added to 40 ml YPDE medium after 24 h to capture the (-)-α-bisabolol. The synthetic SC-URA solid medium (20 g/L glucose, 6.7 g/L yeast nitrogen base without amino acids, and 2.5% agar) was used to screen yeast transformants. YPD medium supplemented with 1.2 g/L 5-fluoroorotic acid and 20 g/L agar was used to remove the *URA3* counter-selection marker (Gao et al., 2017).

### Construction of Building Block Plasmids and Backbone Plasmids

The sequence of (-)-α-bisabolol synthase (*MrBBS*, AIG92846.1) from *Matricaria recutita* (Son et al., 2014) was codon-optimized for *Y. lipolytica* and synthesized by Tsingke Biotech Co., Ltd. Building blocks including promoters, coding genes, and terminators were amplified from the *Y. lipolytica* genome and then cloned into plasmid pUC-T7-FCC1 (Shi et al., 2019). Each building block in the building block plasmids was flanked with *Bsa*I recognition sites and predesigned 4-bp overhangs, in which the internal *Bsa*I recognition sites have been removed by synonymous mutations. The backbone plasmid PGGYL1-3 was derived from pUC19, in which (1) the ampicillin resistance gene was substituted with a kanamycin resistance gene, (2) the internal *Bsa*I site in pUC19 was removed by loop PCR, and (3) *lacZ* flanked with *Bsa*I and 4-bp overhangs was cloned into pUC19. All building block plasmids and backbone plasmids were constructed using the ClonExpress^®^ MultiS One Step Cloning Kit (Vazyme Biotech Co., Ltd., Nanjing, China). All plasmids used in this study and the sequences of building blocks and backbone plasmids are listed in Supplementary Tables S1, S2.

### Yeast Transformation and Strain Construction

The Frozen-EZ Yeast Transformation II Kit was used for *Y. lipolytica* transformation (Shi et al., 2021). A flow chart summarizing the construction of *Y. lipolytica* strains is shown in Figure 5B. All *Y. lipolytica* strains constructed in this study are listed in Table 2.

**TABLE 2:** Strains used in this study.

Strains	Genotype or properties	Sources
*E. coli* DH5α	supE44 ΔlacU169 (φ80 lacZ ΔM15) hsdR17 recA1 endA1 gyrA96 thi-1 relA1	Ktsm-life
Po1h	MatA, leu2-270:LEU, ura3-302, xpr2-322, axp-2	Madzak et al. (2004)
LYW1-1∼ LYW1-12	Po1h with integrated fragment of linearized plasmid pGGYL1-HTBX	This work
LYW2-1∼ LYW2-12	LYW1-11 with integrated fragment of linearized plasmid pGGYL3-HTitY-Gi12S-G19L	This work
LYW3-1∼ LYW3-12	LYW2-11 with integrated fragment of linearized plasmid pGGYL3-HT10X-G13M-GIL	This work
LYW4-1∼ LYW4-12	LYW3-5 with integrated fragment of linearized plasmid pGGYL2-HG8M-T20Y	This work
LYW5-1∼ LYW5-12	LYW4-2 with integrated fragment of linearized plasmid pGGYL1-HTitX	This work
LYW6-1∼ LYW6-12	LYW4-2 with integrated fragment of linearized plasmid pGGYL2-HTitX-EtL	This work
LYW7-1∼ LYW7-12	LYW6-3 with integrated fragment of linearized plasmid pGGYL2-HTiBS-TBY	This work

### Golden Gate Assembly Protocol

The Golden Gate assembly mainly relies on the restriction enzyme *Bsa*I (NEB, R3733L) and T4 DNA Ligase (Takara, 2011A; NEB, M0202T; Vazyme, C301-01). The reaction conditions were based on a previously published protocol (Celińska et al., 2017). The backbone and building block plasmids were first digested with *Bsa*I, and the DNA concentration of each assembly piece was measured. Assembly reaction systems of 20 μl contained 100 ng of the linearized backbone plasmid, equimolar amounts of the other assembly pieces, 2 μl of 10× Takara T4 Buffer, 1 μl of *Bsa*I, Takara T4 Ligase, and ddH_2_O. The assembly reaction was performed in a PCR thermocycler as follows: (3 min at 37°C, 4 min at 16°C) × 60 reaction cycles (or 90 and 120), followed by 5 min at 50°C and 5 min at 80°C. After that, 20 μl of the reaction mixture was used to transform 100 μl of *E. coli* DH5α competent cells.

In this study, the quantification of assembly efficiency was carried out on blue and white spot screening plates. Strains with successful ligation products were white in color, but white colonies could also be mis-joined or false positives. Therefore, PCR analysis and Sanger sequencing were required. In brief, 24 white colonies on the plates were picked randomly. Primer F (5′ to 3′: TCT​CCC​CGC​GCG​TTG​GCC​GAT​T) and primer R (5′ to 3′: GTC​TCG​CGC​GTT​TCG​GTG​ATG) were designed to target the outside regions of *Swa*I in the plasmids (Figures 2A, 3A, 4A). First, the PCR results obtained by colony PCR and DNA gel electrophoresis were used to select the possibly positive colonies. Subsequently, the 4-bp overhangs in the assembled plasmids (extracted from possibly positive colonies) were sequenced to rule out the possibility of mis-joined or false positive cases. The final assembly efficiency is the ratio of the number of successfully assembled spots to the total white spots (24 colonies). The quantification of transformation efficiency was the ratio of the number of total white spots on the plates to the linearized backbone plasmid (100 ng).

Finally, the successfully assembled plasmid was linearized by restriction digestion with *Swa*I and used to transform *Y. lipolytica* for random integration.

### Analytical Methods

In order to determine the (-)-α-bisabolol titer, the standard curve was measured by diluting the standard with dodecane to yield concentrations of 0.001, 0.002, 0.004, 0.008, 0.02, 0.04, 0.06, 0.08, 0.10, 0.20, 0.40, 0.60, 0.80, and 1.00 g/L and analyzed by gas chromatography–mass spectrometry. The dodecane phase and the fermentation broth were centrifuged at 5,000 g for 5 min. The upper dodecane phase was filtered through a 0.22-μm-pore-size membrane and analyzed on a QP2020NX instrument (Shimadzu, Japan) equipped with an HP-5MS column (30 m × 320 μm × 0.5 μm). The oven program consisted of a ramp at 20°C/min to 280°C for 20 min, with N_2_ as the carrier gas at a flow rate of 1.14 ml/min. The retention time of (-)-α-bisabolol was 12.24 min. The cell pellet was washed twice with distilled water and lyophilized for further analysis.

### 5-L Fermentation

An inoculum comprising 5 ml of a pre-culture in YPD was first transferred into 45 ml of fresh YPD medium. After 24 h of cultivation at 28°C and 240 rpm, 20 ml of the culture was transferred into 180 ml of YPD in a 500-ml shake flask and cultivated under the same conditions for 18 h. Then, 200 ml of the resulting seed culture was used to inoculate a 5-L bioreactor (T&J-Atype, Shanghai, China) containing 2 L of medium for fed-batch fermentation. The initial fermentation medium consisted of 60 g/L glucose, 40 g/L peptone, and 20 g/L yeast extract. The temperature, dissolved oxygen, and agitation speed were maintained at 30°C, 1 vvm, and 600 rpm, respectively. The pH was controlled at 7.0 by automatically adding 3 M potassium hydroxide or 3 M sulfuric acid. When the glucose concentration fell below 2 g/L, feed solution containing 800 g/L glucose was added to reach a glucose concentration of 80 g/L in the bioreactor. The (-)-α-bisabolol titer, glucose content, and OD_600_ were measured every 12 h.

## Results and Discussion

### Preparing the Modular Parts for Golden Gate Assembly

NHEJ was recently found to be a promising method for chromosomal integration in *Y. lipolytica* (Cui et al., 2021). However, there is currently no modular assembly tool for the construction of multi-gene biosynthetic pathways applied for NHEJ integration, which makes genetic manipulation time-consuming and laborious. Therefore, we constructed a Golden Gate library for terpene production in *Y. lipolytica*.

**FIGURE 1:**
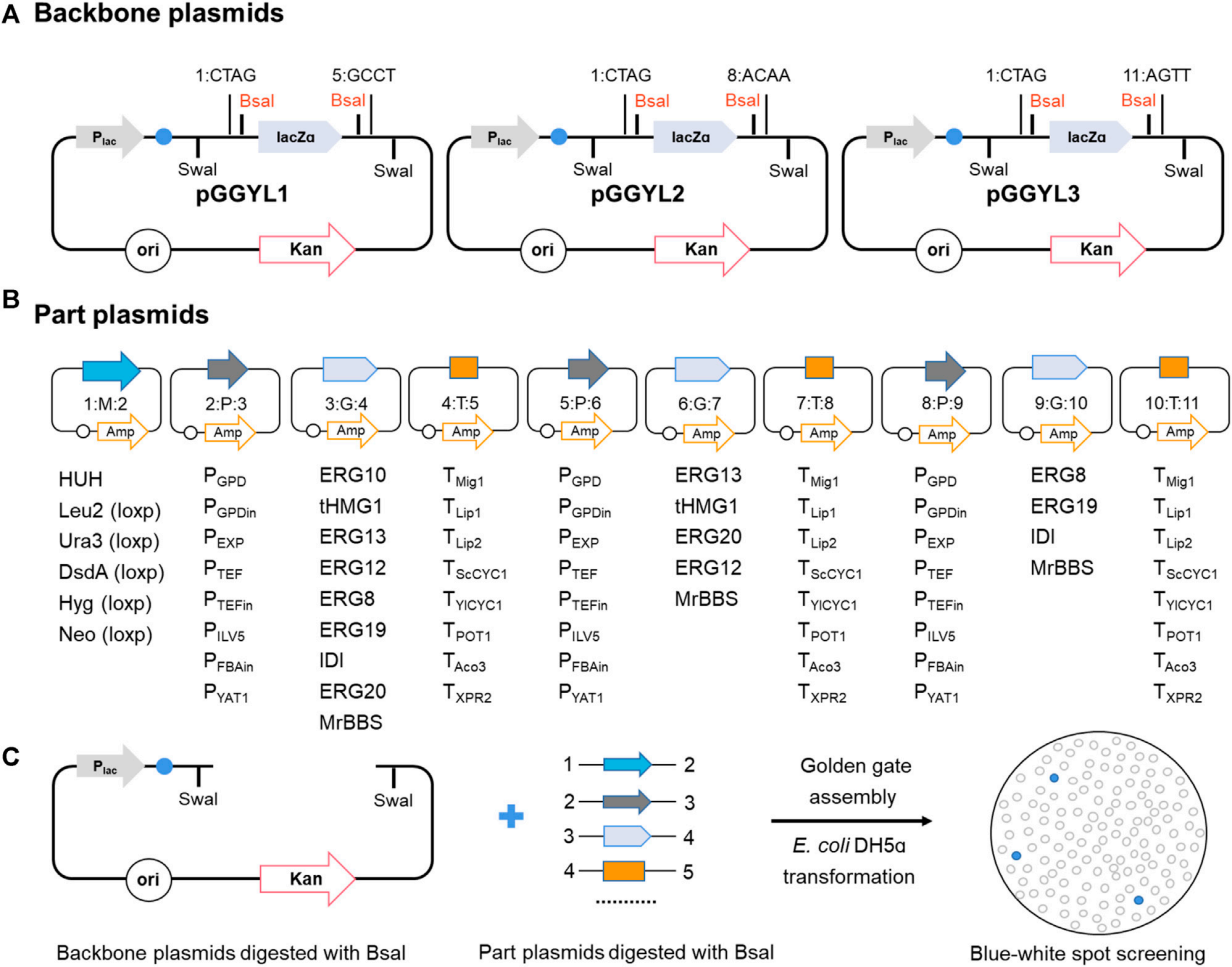
Non-homologous end-joining (NHEJ) integration-based Golden Gate cloning toolkit dedicated to *Yarrowia lipolytica*. **(A)** The backbone plasmid pGGYL1-3 was designed for the assembly of 4, 7, and 10 DNA fragments covering 1–3 transcription units, respectively. *Swa*I recognition sites were included to cut the assembled selection marker and transcription unit for *Y. lipolytica* transformation. **(B)** Building blocks, including the selection marker, promoters, coding gene, and terminator, were cloned into pUC57-T7-FCC1 to generate building block plasmids. The building blocks were flanked with predesigned 4-bp overhangs. The *Bsa*I cleavage sequences at the junction sites of two building blocks were designed to be complementary with each other (each assigned a number from 1 to 11). **(C)** Schematic diagram of the NHEJ integration-based Golden Gate cloning toolkit. The backbone and building block plasmids were digested with *Bsa*I, assembled in one step, and used to transform *Escherichia coli* DH5ɑ. White colonies were picked for verification.

The library consists of a total of 75 backbone plasmids and the building block plasmids. Three backbone plasmids—pGGYL1, pGGYL2, and pGGYL3—can be used for the ligation of 4, 7, and 10 fragments, respectively (Figure 1A). In order to make the subsequent transformant screening more convenient, a lacZ expression box was inserted between the *Bsa*I sites in the pGGYL1-3 plasmid for blue–white colony screening.

The 72 building block plasmids contain genetic parts, such as selection markers, promoters, protein-coding gene sequences, and terminators. Each building block is flanked with respective *Bsa*I recognition sites and predesigned 4-bp overhangs (from 1 to 11). The *Bsa*I cleavage sequences at the junction sites of two building blocks are designed to be complementary with each other. As shown in Figure 1B, eight endogenous promoters (P_GPD_, P_GPDin_, P_EXP_, P_TEF_, P_TEFin_, P_ILV5_, P_FBAin_, and P_YAT_) and nine terminators (T_Mig1_, T_Lip1_, T_Lip2_, T_ScCYC1_, T_YICYC1_, T_TEF_, T_POT1_, T_Aco3_, and T_XPR2_) with different transcriptional and termination strengths in *Y. lipolytic*a were selected in order to regulate the gene expression effectively (Curran et al., 2015; Dulermo et al., 2017; Holkenbrink et al., 2018). Eight genes (*tHMG1*, *IDI*, *ERG8*, *ERG10*, *ERG12*, *ERG13*, *ERG19*, and *ERG20*) from the mevalonate (MVA) pathway were selected as building blocks to enhance the terpene biosynthesis flux. Six selection markers, including *hisG-URA3-hisG* (*HUH*), *LEU2* (β-isopropylmalate dehydrogenase), *HPH* (hygromycin B phosphotransferase), *NEO* (aminoglycoside phosphotransferase), *URA3* (orotidine-5′-phosphate decarboxylase), and *DsdA* (D-serine ammonia-lyase), were selected to be used in different auxotrophic or non-auxotrophic strains of *Y. lipolytica*. Among these, *LEU*, *HPH*, *NEO*, *URA3*, and *DsdA* were flanked with loxp tags (locus of X cross-over in P1) to make the markers recyclable. Furthermore, to ensure efficient assembly, the internal *Bsa*I and *Swa*I recognition sites inside the building blocks were removed by introducing synonymous mutations. After the assembly reaction was finished and the DNA was introduced into *E. coli* DH5ɑ, white colonies were screened for identification of successfully assembled plasmid by multiplex PCR and Sanger sequencing (Figure 1C).

The successfully constructed plasmid can be digested with *Swa*I. After the target fragment is recovered, it can be used to transform *Y. lipolytica* for random integration.

### Optimization of the Assembly Efficiency

It was previously reported that the assembly efficiency decreased with an increasing number of ligation fragments (Marillonnet and Werner, 2015). Therefore, the DNA ligation conditions were subsequently optimized to maximize the assembly efficiency of 4, 7, and 10 fragments.

**FIGURE 2:**
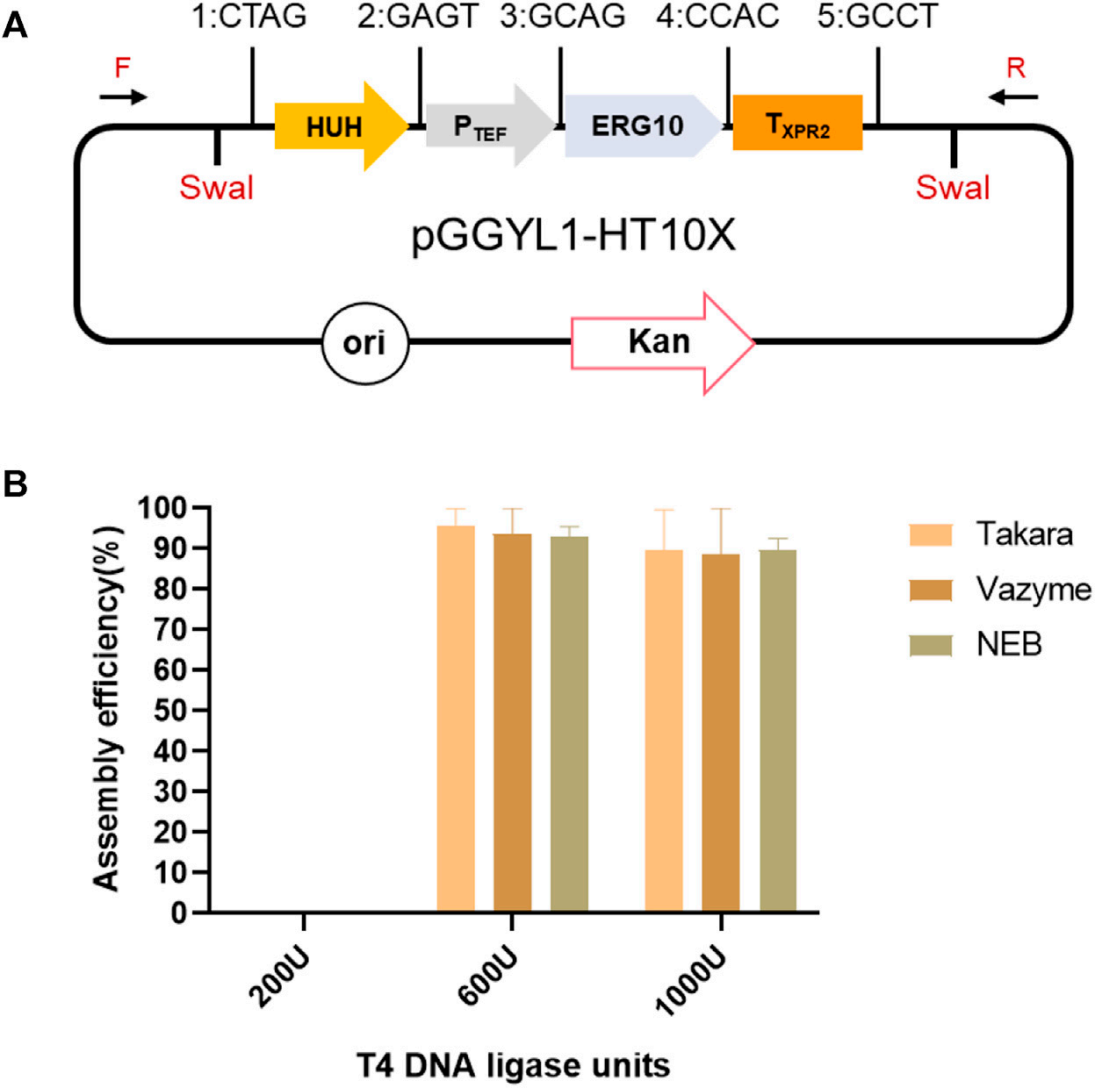
Assembly of four fragments using YALIcloneNHEJ. **(A)** Schematic representation of the completely assembled plasmid pGGYL1-HT10X. **(B)** Assembly efficiency using different amounts of T4 DNA ligase from three commercial suppliers. The primers F/R were used to amplify the assembled fragments.

Considering that T4 DNA ligase has a direct effect on assembly efficiency (Potapov et al., 2018), T4 ligases from three different producers (Takara, NEB, and Vazyme) were selected, and their assembly efficiency was compared when four fragments (*HUH*, *P*_*TEF*_, *ERG10*, and *T*_*XPR2*_) were assembled (Figure 2A). When 200 U of enzyme was used, no successful assembly cases were found, indicating that the enzyme amount was too low to support the ligation reaction. Therefore, the T4 DNA ligase amount was increased to 600 and 1,000 U, respectively. It was found that the assembly efficiency of four fragments could reach more than 90% with all three enzymes (Figure 2B). Taking into account the lower price of T4 DNA ligase from Takara, this enzyme was used for further experiments.

**FIGURE 3:**
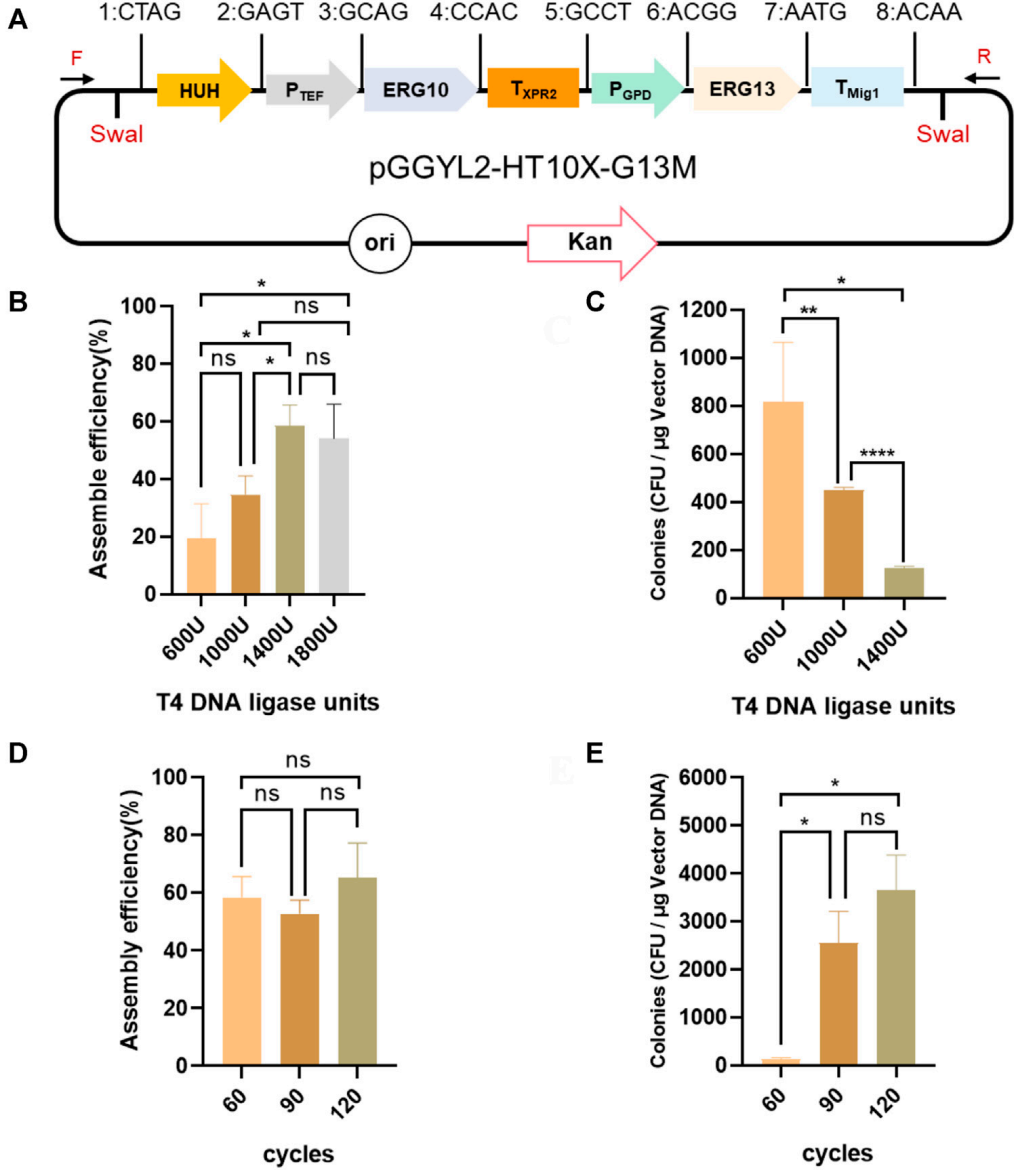
Assembly of seven fragments using YALIcloneNHEJ. **(A)** Schematic representation of the completely assembled plasmid pGGYL2-HT10X-G13M. **(B)** The effect of different amounts of Takara T4 DNA ligase on the assembly efficiency of seven DNA fragments. **(C)** The effect of different amounts of Takara T4 DNA ligase on the number of transformants. **(D)** Assembly efficiency using different numbers of reaction cycles with 1,400 U of T4 DNA ligase. **(E)** Number of transformants using different numbers of reaction cycles with 1,400 U of T4 DNA ligase. The primer F/R was used to amplify the assembled fragments.

Subsequently, the assembly efficiency of seven fragments (*HUH*, *P*_*TEF*_, *ERG10*, *T*_*XPR2*_, *P*_*GPD*_, *ERG13*, and *T*_*Mig1*_) was verified under the same conditions (Figure 3A). However, the assembly efficiency reached only 19.4 ± 9.8%, indicating that 600 U of T4 DNA ligase may not be enough for the assembly of seven fragments (Figure 3B). Therefore, the ligase amounts were increased to 1,000 and 1,400 U. It was found that the assembly efficiency was improved with increased ligase amounts, and the highest reached 58.5 ± 5.2%. Therefore, the ligase amounts were further increased to 1,800 U, but there was no further increase of efficiency (Figure 3B). In addition, although the assembly efficiency was highest with 1,400 U of T4 ligase, it came at the cost of a decreased number of transformants (Figure 3C). Consequently, it was significant to search for an approach to balance the assembly efficiency and the number of transformants. By increasing the number of ligation cycles with 1,400 U of T4 ligase from the original 60 cycles to 90 and 120 cycles, it was found that, although the assembly efficiency was not be improved (Figure 3D), the number of transformants increased a remarkable 20 times from 133 ± 21 to 3,655 ± 515 cfu/μg vector (Figure 3E). Due to the fact that the transformation efficiency of circular plasmid is dozens of times higher than that of linear plasmid in *E. coli*, we thus speculated that when the number of ligation cycles was increased, multigene fragments (which could not be assembled as a circular plasmid in a short time) assembled successfully in an extended period of time. Therefore, the number of transformants was improved significantly. In brief, increasing the number of ligation cycles can be a good strategy for improving the efficiency of Golden Gate assembly.

**FIGURE 4:**
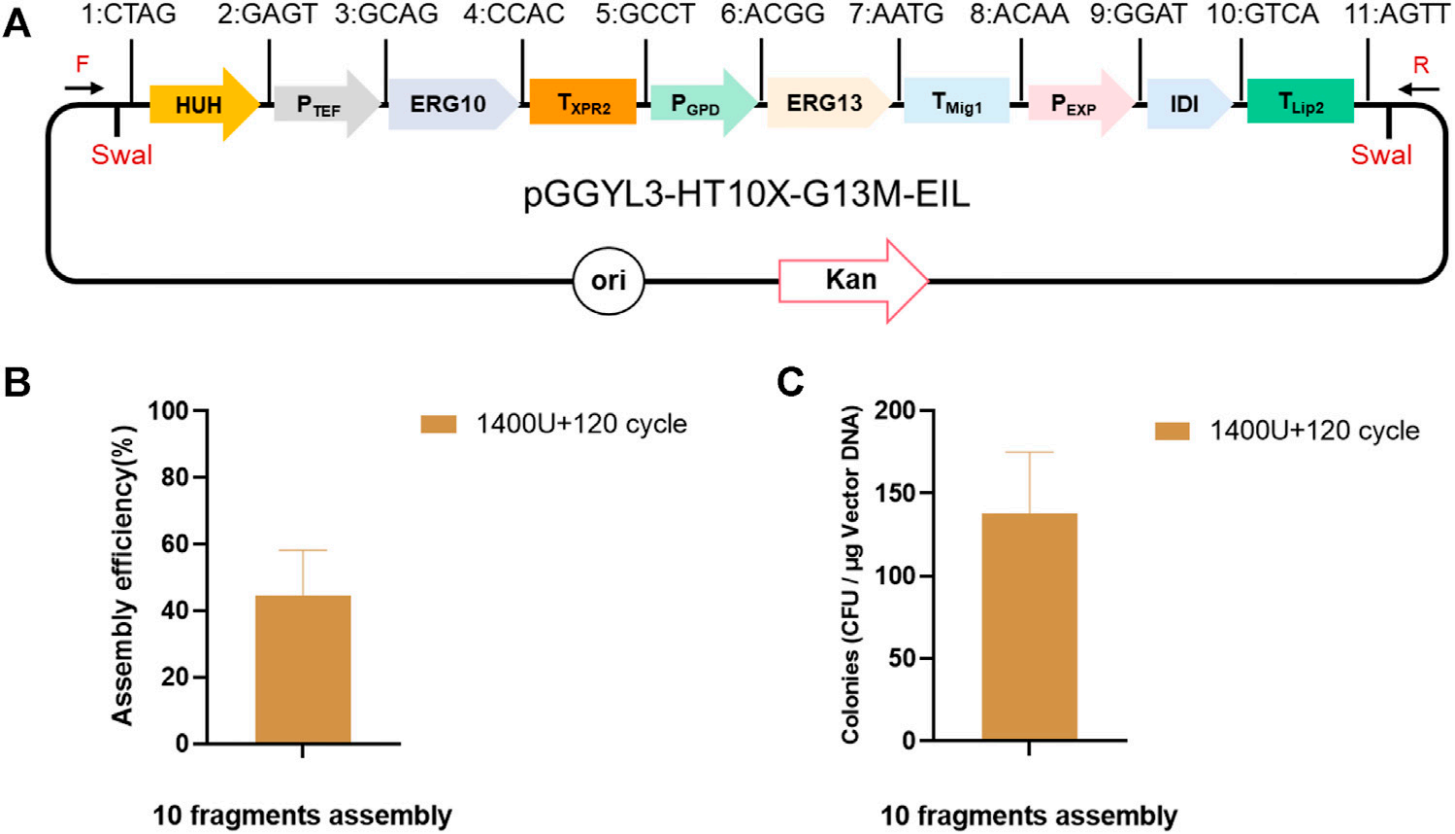
Assembly of 10 fragments using YALIcloneNHEJ. **(A)** Schematic representation of the completely assembled plasmid pGGYL3-HT10X-G13M-EIL. **(B)** Assembly efficiency with 1,400 U of T4 DNA ligase and 120 reaction cycles. **(C)** Number of transformants with 1,400 U of T4 DNA ligase and 120 reaction cycles. The primer F/R was used to amplify the assembled fragments.

Finally, the optimized ligation conditions (1,400 U, 120 cycles) were used for the assembly of 10 fragments (*HUH*, *P*_*TEF*_, *ERG10*, *T*_*XPR2*_, *P*_*GPD*_, *ERG13*, *T*_*Mig1*_, *P*_*EXP*_, *IDI*, and *T*_*Lip2*_), and the assembly length reached 13.8 kb (Figure 4A). In this case, the number of transformants still reached 1,380 ± 30 cfu/μg vector, with more than 44.5 ± 11.2% assembly efficiency (Figures 4B,C), which was high enough for plasmid assembly and verification.

These results demonstrate that, with careful optimization of ligation conditions, Golden Gate can efficiently assemble 10 DNA fragments *via* a simple one-step ligation reaction, which achieved the simultaneous assembly and expression of three genes.

### Constructing a Baseline (-)-α-Bisabolol Production Strain by Randomly Integrating the *MrBBS* Cassette

**FIGURE 5:**
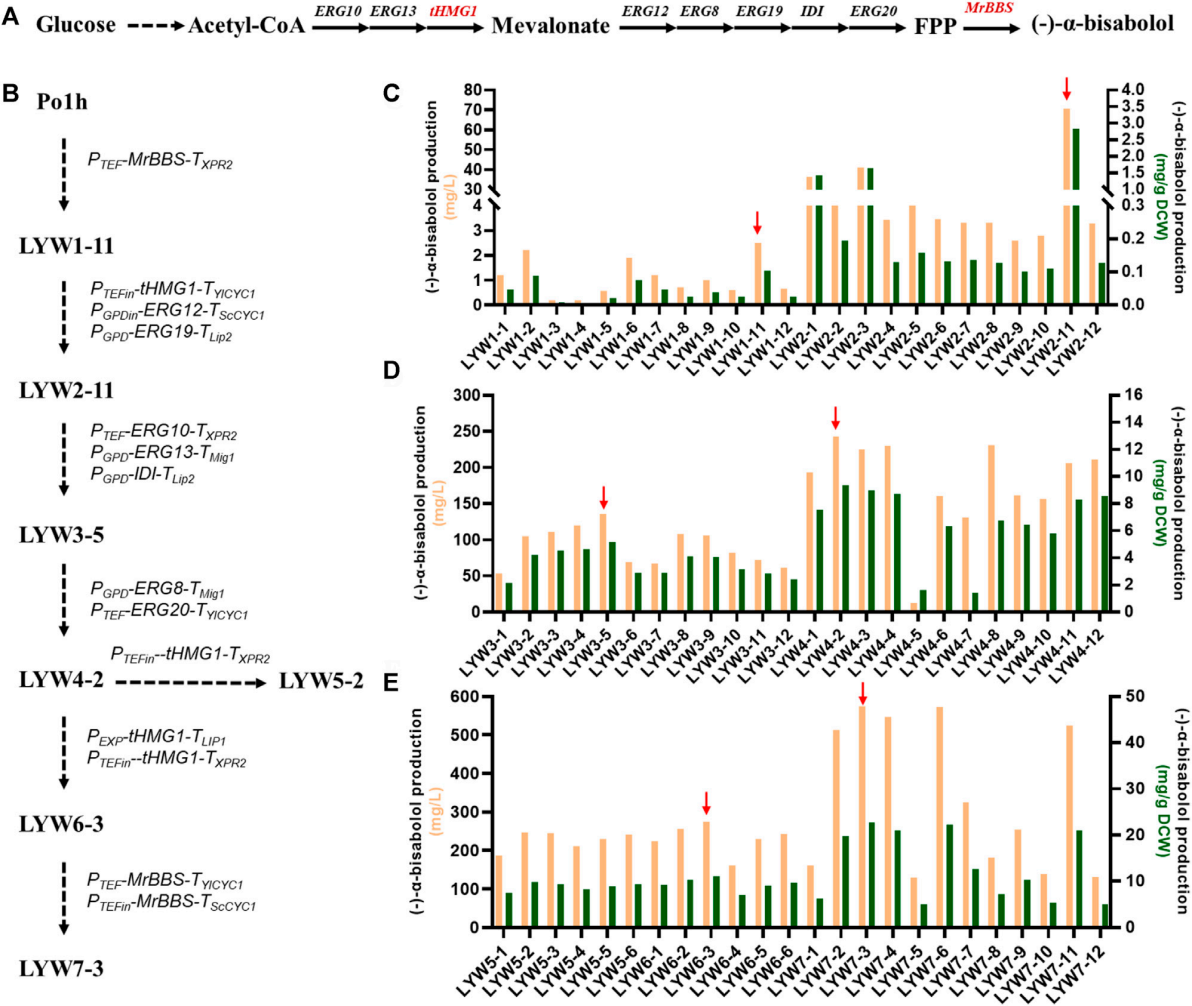
Overproduction of (-)-α-bisabolol by optimizing the mevalonate pathway. **(A)** (-)-α-Bisabolol biosynthesis pathway. **(B)** Flow chart describing the construction of engineered *Yarrowia lipolytica* strains. **(C)** (-)-α-Bisabolol titer after randomly integrating *MrBBS* into Po1h and *tHMG1* as well as *ERG12* and *ERG19* into LYW1-11. **(D)** (-)-α-Bisabolol titer after randomly integrating *ERG10*, *ERG13*, and *IDI* into LYW2-11 and *ERG8* and *ERG20* into LYW3-5. **(E)** (-)-α-Bisabolol titer after randomly integrating a single copy or two copies of *tHMG1* into LYW4-2 and two copies of *MrBBS* into LYW6-3. The red arrow indicates the best-performing strain.

After the Golden Gate library for NHEJ integration was successfully constructed, we sought to use the library to quickly construct an (-)-α-bisabolol-overproducing strain.

(-)-α-Bisabolol is synthesized by *MrBBS* from the precursor farnesyl diphosphate (FPP), which is an intermediate of the MVA pathway (Figure 5A) (Sarrade-Loucheur et al., 2020; Kim et al., 2021). Therefore, we first verified whether *MrBBS* was functional in *Y. lipolytica.* We chose the strain Po1h derived from W29 as the host (Nicaud et al., 2002; Madzak et al., 2004). Subsequently, the plasmid pGGYL1-HTBX containing the selection marker *HUH* and *MrBBS* expression cassette was constructed using YALIcloneNHEJ. After the plasmid was linearized by digestion with *Swa*I, the *HUH* and *MrBBS* expression cassettes were recovered and used to transform *Y. lipolytica* protoplasts for random chromosomal integration. Twelve transformants (designated LYW1-1 to LYW1-12) were picked randomly to test the yield of (-)-α-bisabolol after 4 days of fermentation. It was found that all picked strains were able to produce (-)-α-bisabolol, indicating 100% integration efficiency (Figure 5C). Among these, the highest titer of strain LYW1-11 reached 2.5 mg/L, corresponding to a yield of 0.103 mg/g dry cell weight (DCW). Therefore, the LYW1-11 strain was selected for further optimization.

### Improving the (-)-α-Bisabolol Titer by Iteratively Integrating an Entire MVA Pathway

Although (-)-α-bisabolol could be synthesized heterologously in *Y. lipolytica*, the titer was still too low to meet the demands of industrial production. Previous studies have demonstrated that enhancement of the MVA pool favors the accumulation of related terpene products (Yang et al., 2016; Liu et al., 2020). Therefore, eight genes in the MVA pathway for the synthesis of FPP from acetyl-CoA, including *ERG10*, *ERG13*, truncated *HMG1* (*tHMG1*), *ERG12*, *ERG8*, *ERG19*, *IDI*, and *ERG20*, were overexpressed to push the carbon flux from acetyl-CoA towards (-)-α-bisabolol. The eight genes were placed under the control of a series of strong constitutive promoters and divided into three sets by Golden Gate assembly: (1) *P*_*TEFin*_-*tHMG1*-*T*_*YlCYC1*_, *P*_*GPDin*_-*ERG12*-*T*_*ScCYC1*_, and *P*_*GPD*_ *-ERG19-T*_*Lip2*_, (2) *P*_*TEF*_ *-ERG10-T*_*XPR2*_, *P*_*GPD*_ *-ERG13-T*_*Mig1*_, and *P*_*GPD*_-*IDI*-*T*_*Lip2*_, and (3) *P*_*GPD*_ *-ERG8-T*_*Mig1*_ and *P*_*TEF*_ *-ERG20-T*_*YlCYC*1_. Subsequently, the three sets were iteratively integrated into strain LYW1-11, and 12 transformants were randomly picked after each round of integration to test the (-)-α-bisabolol titer. The best-performing strains were selected for the next iterative round of transformation.

As shown in Figure 5C, LYW2-11 produced the highest (-)-α-bisabolol titer among 12 randomly picked strains (70.6 mg/L, 2.83 mg/g DCW). When *tHMG1*, *ERG12*, and *ERG19* were first introduced to LYW1-11, the titer of the resulting strain was 28-fold higher compared with the control strain LYW1-1. After that, *ERG10*, *ERG13*, and *IDI* were integrated into the best-performing strain LYW2-11. The (-)-α-bisabolol titer among the eight picked strains in this round exceeded that of LYW2-11, and the highest (-)-α-bisabolol titer of LYW3-5 can reach 135.3 mg/L (5.16 mg/g DCW), a 91.6% increase compared with the parental strain (Figure 5D). Based on this excellent result, the last two genes of the MVA pathway, *ERG8* and *ERG20*, were integrated into LYW3-5. The best-performing strain LYW4-2 produced 242.6 mg/L (9.34 mg/g DCW) (-)-α-bisabolol, a further 79.3% increase compared with LYW3-5 (Figure 5D).

Following three rounds of random integration of an entire MVA pathway, the (-)-α-bisabolol titer was increased from 2.5 to 242.6 mg/L, a nearly 97-fold improvement, which indicated that dredging the MVA pool by NHEJ-based random integration was an effective strategy for (-)-α-bisabolol overproduction. This method could also be applied to the production of other terpenoids in *Y. lipolytica* in the future.

### Optimizing the Copy Number of the Cassettes Encoding the Rate-Limiting Enzymes *tHMG1* and *MrBBS*

In terpenoid biosynthesis, *tHMG1* is generally considered a key rate-limiting enzyme which irreversibly reduces 3-hydroxy-3-methyl glutaryl coenzyme A to mevalonate (Gao et al., 2017). Therefore, *tHMG1* was first overexpressed. A single copy of *tHMG1* and two copies of *tHMG1* under the control of strong promoters were individually introduced into strain LYW4-2. Six strains (designated LYW5-1 to LYW5-6 and LYW6-1 to LYW6-6) were respectively picked, but none showed a significantly improved (-)-α-bisabolol. The best-performing strain LYW6-3 could produce 275.3 mg/L (11.03 mg/g DCW) (-)-α-bisabolol, which was 13.48% higher than the titer of LYW4-2 (Figure 5E). These results indicated that the expression level of *tHMG1* was high enough, and this reaction was no longer a limiting step. Terpenoid synthases are also generally considered key rate-limiting enzymes because they directly convert the precursor skeleton into various terpenoid products (Li et al., 2021). Thus, in order to increase the titer, two copies of *MrBBS* under the control of strong promoters were introduced into strain LYW6-3. It was found that 6 of 12 randomly picked transformations produced higher (-)-α-bisabolol titers than the control strain LYW6-3. In particular, strain LYW7-3 produced 573.7 mg/L (22.71 mg/g DCW) (-)-α-bisabolol in shake flasks, which was a 108.4% improvement over LYW6-3 (Figure 5E). To our best knowledge, this is the highest shake-flask titer of (-)-α-bisabolol reported in yeast so far.

In addition, the stability of the selected strains used for the next round of transformation, including LYW1-11, LYW2-11, LYW3-5, LYW4-2, LYW5-2, LYW6-3, and the final optimized strain LYW7-3, was also investigated. After 10 generations of continuous subculture, the (-)-α-bisabolol titer was not significantly lower than that of the first generation, which indicated that the engineered strains were genetically stable (Supplementary Figure S1).

In summary, terpenoid production could be significantly improved by dredging the MVA pathway. In addition, a mutant library can be quickly constructed based on NHEJ integration to screen hyperproducer strains.

### High-Density Fermentation

**FIGURE 6:**
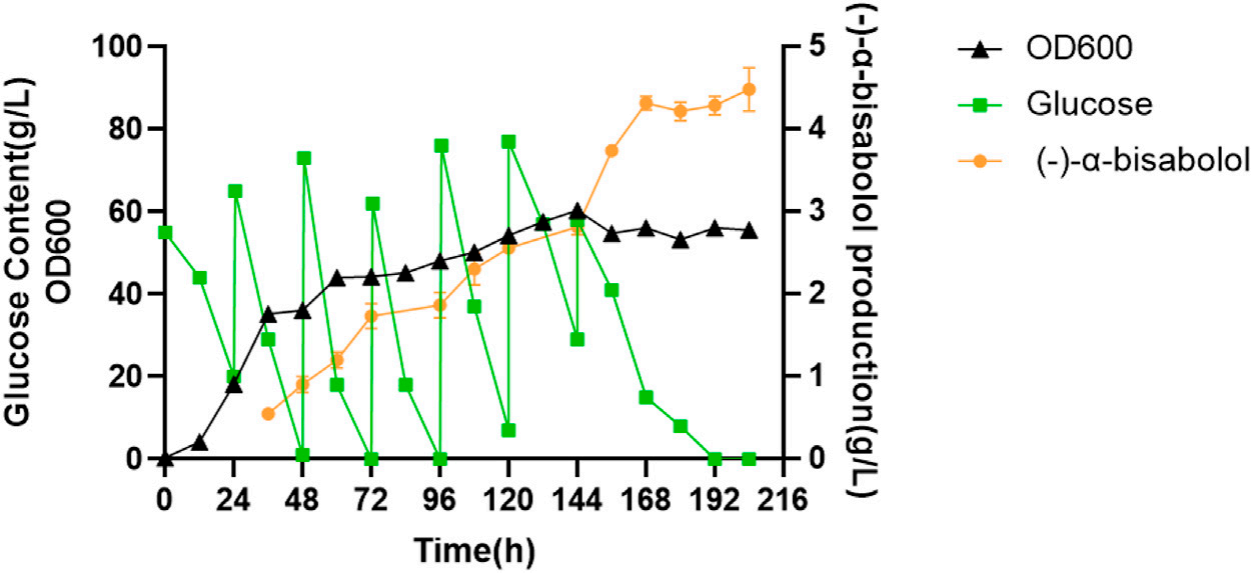
Fed-batch bioreactor fermentation for (-)-α-bisabolol production.

In order to explore the potential of LYW7-3 for large-scale production of (-)-α-bisabolol, we carried out fed-batch fermentation experiments in a 5-L bioreactor. As shown in Figure 6, cell growth entered the stationary phase at about 60 h. After that, the cell growth was relatively slow, and the highest OD_600_ value reached 60.40 after 144 h of fermentation. However, (-)-α-bisabolol increased continuously, reaching 4.4 g/L after 168 h, which corresponds to a productivity of 26.2 mg L^−1^ h^−1^. After that, the titer remained basically unchanged. To the best of our knowledge, this is the highest (-)-α-bisabolol titer reported in yeast to date. These results showed that terpenoid biosynthesis could be boosted through high-density fermentation. What is more, engineering *Y. lipolytica* for terpenoid production quickly by using an efficient NHEJ integration-based modular cloning toolkit is a powerful new strategy, which can be used for a highly efficient production of various terpenoids in the near future.

## Conclusion

In this study, we build a Golden Gate cloning toolkit YALIcloneNHEJ, which could assemble 4, 7, and 10 fragments simultaneously with high efficiency in one step. Using this toolkit, we constructed a (-)-α-bisabolol biosynthesis route in *Y. lipolytica*. After randomly integrating an entire MVA pathway and optimizing the copy number of rate-limiting enzymes *tHMG1* and *MrBBS*, the (-)-α-bisabolol titer in the resulting strain LYW7-3 reached 4.4 g/L, the highest (-)-α-bisabolol titer reported in yeast. Our study expands the toolbox for metabolic engineering in *Y. lipolytica* and is expected to be used for a highly efficient production of various terpenoids since all plasmids can be shared by the community.

## Data Availability

The original contributions presented in the study are included in the article/Supplementary Material. Further inquiries can be directed to the corresponding authors.

## Abbreviations

ERG10, acetoacetyl-CoA thiolase; ERG13, hydroxymethylglutaryl-CoA synthase; tHMG1, truncated hydroxymethylglutaryl-CoA reductase; ERG12, mevalonate kinase; ERG8, phosphomevalonate kinase; ERG19, mevalonate diphosphate decarboxylase; IDI, isopentenyl diphosphate isomerase; ERG20, geranyl/farnesyl diphosphate synthase; PCR, polymerase chain reaction.
