# Supplementary material for: YALIcloneNHEJ: An Efficient Modular Cloning Toolkit for NHEJ Integration of Multigene Pathway and Terpenoid Production in Yarrowia lipolytica
Source: Front Bioeng Biotechnol. 2022 Mar 2;9:816980. doi: 10.3389/fbioe.2021.816980 (PMC8924588; doi:10.3389/fbioe.2021.816980)
Supplement: Supplementary file 1 [file DataSheet1.docx]

# Supporting Information for

**YALIcloneNHEJ: an efficient modular cloning toolkit for NHEJ integration of multigene pathway and terpenoid production in** ***Yarrowia lipolytica***

Ya-Wen Li^1^, Cai-Ling Yang^1^, Qi Shen^1^, Qian-Qian Peng^1^, Qi Guo^2^, Zhi-Kui Nie^3^, Xiao-Man Sun^1^, Tian-Qiong Shi^1,^*, Xiao-Jun Ji^2,^*, He Huang^1,4^

^1^ School of Food Science and Pharmaceutical Engineering, Nanjing Normal University, 2 Xuelin Road, Qixia District, Nanjing, 210023, People’s Republic of China

^2^ College of Biotechnology and Pharmaceutical Engineering, Nanjing Tech University, No. 30 South Puzhu Road, Nanjing 211816, People’s Republic of China

^3^ Jiangxi New Reyphon Biochemical Co., Ltd, Salt & Chemical Industry, Xingan, Jiangxi, 331399, People’s Republic of China

^4^ College of Pharmaceutical Sciences, Nanjing Tech University, No. 30 South Puzhu Road, Nanjing 211816, People’s Republic of China

***Corresponding Author**: Tian-Qiong Shi, [tqshi@njnu.edu.cn](mailto:tqshi@njnu.edu.cn); Xiao-Jun Ji, [xiaojunji@njtech.edu.cn](mailto:xiaojunji@njtech.edu.cn);

**Table S1 Plasmids used in this study**

| **plasmids** | **Genotype or properties** | **Sources** |
| --- | --- | --- |
| pUC19 | Clone vector, AmpR | Shi et al., 2019 |
| pUC57-T7-FCC1 | Clone vector, AmpR | Shi et al., 2019 |
| pGGYL1 | KanR, lacZ flanked with BsaI and predesigned 4-bp overhangs (1 and 5) | This work |
| pGGYL2 | KanR, lacZ flanked with BsaI and predesigned 4-bp overhangs (1 and 8) | This work |
| pGGYL3 | KanR, lacZ flanked with BsaI and predesigned 4-bp overhangs (1 and 11) | This work |
| pUC-12-HUH | AmpR, hisG-URA3-hisG with BsaI and predesigned 4-bp overhangs (1 and 2) | This work |
| pUC-12-LEU2loxp | AmpR, loxp-LEU2-loxp with BsaI and predesigned 4-bp overhangs (1 and 2) | This work |
| pUC-12-URA3loxp | AmpR, loxp-URA3-loxp with BsaI and predesigned 4-bp overhangs (1 and 2) | This work |
| pUC-12-DsdAloxp | AmpR, loxp-P_TEF_-DsdA-T_XPR2_-loxp with BsaI and predesigned 4-bp overhangs (1 and 2) | This work |
| pUC-12-Hygloxp | AmpR, loxp-P_TEF_-Hyg-T_XPR2_-loxp with BsaI and predesigned 4-bp overhangs (1 and 2) | This work |
| pUC-12-Neoloxp | AmpR, loxp-P_TEF_-Neo-T_XPR2_-loxp with BsaI and predesigned 4-bp overhangs (1 and 2) | This work |
| pUC-23-GPD | AmpR, GPD promoter flanked with BsaI and predesigned 4-bp overhangs (2 and 3) | This work |
| pUC-23-GPDin | AmpR, GPDin promoter flanked with BsaI and predesigned 4-bp overhangs (2 and 3) | This work |
| pUC-23-EXP | AmpR, EXP promoter flanked with BsaI and predesigned 4-bp overhangs (2 and 3) | This work |
| pUC-23-TEF | AmpR, TEF promoter flanked with BsaI and predesigned 4-bp overhangs (2 and 3) | This work |
| pUC-23-TEFin | AmpR, TEFin promoter flanked with BsaI and predesigned 4-bp overhangs (2 and 3) | This work |
| pUC-23-ILV5 | AmpR, ILV5 promoter flanked with BsaI and predesigned 4-bp overhangs (2 and 3) | This work |
| pUC-23-FBAin | AmpR, FBAin promoter flanked with BsaI and predesigned 4-bp overhangs (2 and 3) | This work |
| pUC-23-YAT1 | AmpR, YAT1 promoter flanked with BsaI and predesigned 4-bp overhangs (2 and 3) | This work |
| pUC-34-ERG10 | AmpR, ERG10 gene flanked with BsaI and predesigned 4-bp overhangs (3 and 4) | This work |
| pUC-34-tHMG1 | AmpR, tHMG1 gene flanked with BsaI and predesigned 4-bp overhangs (3 and 4) | This work |
| pUC-34-ERG13 | AmpR, ERG13 gene flanked with BsaI and predesigned 4-bp overhangs (3 and 4) | This work |
| pUC-34-ERG12 | AmpR, ERG12 gene flanked with BsaI and predesigned 4-bp overhangs (3 and 4) | This work |
| pUC-34-ERG8 | AmpR, ERG8 gene flanked with BsaI and predesigned 4-bp overhangs (3 and 4) | This work |
| pUC-34-ERG19 | AmpR, ERG19 gene flanked with BsaI and predesigned 4-bp overhangs (3 and 4) | This work |
| pUC-34-IDI | AmpR, IDI gene flanked with BsaI and predesigned 4-bp overhangs (3 and 4) | This work |
| pUC-34-ERG20 | AmpR, ERG20 gene flanked with BsaI and predesigned 4-bp overhangs (3 and 4) | This work |
| pUC-34-MrBBS | AmpR, MrBBS gene flanked with BsaI and predesigned 4-bp overhangs (3 and 4) | This work |
| pUC-45-Mig1 | AmpR, Mig1 terminator flanked with BsaI and predesigned 4-bp overhangs (4 and 5) | This work |
| pUC-45-Lip1 | AmpR, Lip1 terminator flanked with BsaI and predesigned 4-bp overhangs (4 and 5) | This work |
| pUC-45-Lip2 | AmpR, Lip2 terminator flanked with BsaI and predesigned 4-bp overhangs (4 and 5) | This work |
| pUC-45-ScCYC1 | AmpR, ScCYC1 terminator flanked with BsaI and predesigned 4-bp overhangs (4 and 5) | This work |
| pUC-45-YlCYC1 | AmpR, YlCYC1 terminator flanked with BsaI and predesigned 4-bp overhangs (4 and 5) | This work |
| pUC-45-POT1 | AmpR, POT1 terminator flanked with BsaI and predesigned 4-bp overhangs (4 and 5) | This work |
| pUC-45-Aco3 | AmpR, Aco3 terminator flanked with BsaI and predesigned 4-bp overhangs (4 and 5) | This work |
| pUC-45-XPR2 | AmpR, XPR2 terminator flanked with BsaI and predesigned 4-bp overhangs (4 and 5) | This work |
| pUC-56-GPD | AmpR, GPD promoter flanked with BsaI and predesigned 4-bp overhangs (5 and 6) | This work |
| pUC-56-GPDin | AmpR, GPDin promoter flanked with BsaI and predesigned 4-bp overhangs (5 and 6) | This work |
| pUC-56-EXP | AmpR, EXP promoter flanked with BsaI and predesigned 4-bp overhangs (5 and 6) | This work |
| pUC-56-TEF | AmpR, TEF promoter flanked with BsaI and predesigned 4-bp overhangs (5 and 6) | This work |
| pUC-56-TEFin | AmpR, TEFin promoter flanked with BsaI and predesigned 4-bp overhangs (5 and 6) | This work |
| pUC-56-ILV5 | AmpR, ILV5 promoter flanked with BsaI and predesigned 4-bp overhangs (5 and 6) | This work |
| pUC-56-FBAin | AmpR, FBAin promoter flanked with BsaI and predesigned 4-bp overhangs (5 and 6) | This work |
| pUC-56-YAT1 | AmpR, YAT1 promoter flanked with BsaI and predesigned 4-bp overhangs (5 and 6) | This work |
| pUC-67-tHMG1 | AmpR, tHMG1 gene flanked with BsaI and predesigned 4-bp overhangs (6 and 7) | This work |
| pUC-67-ERG13 | AmpR, ERG13 gene flanked with BsaI and predesigned 4-bp overhangs (6 and 7) | This work |
| pUC-67-ERG12 | AmpR, ERG12 gene flanked with BsaI and predesigned 4-bp overhangs (6 and 7) | This work |
| pUC-67-ERG20 | AmpR, ERG20 gene flanked with BsaI and predesigned 4-bp overhangs (6 and 7) | This work |
| pUC-67-MrBBS | AmpR, MrBBS gene flanked with BsaI and predesigned 4-bp overhangs (6 and 7) | This work |
| pUC-78-Mig1 | AmpR, Mig1 terminator flanked with BsaI and predesigned 4-bp overhangs (7 and 8) | This work |
| pUC-78-Lip1 | AmpR, Lip1 terminator flanked with BsaI and predesigned 4-bp overhangs (7 and 8) | This work |
| pUC-78-Lip2 | AmpR, Lip2 terminator flanked with BsaI and predesigned 4-bp overhangs (7 and 8) | This work |
| pUC-78-ScCYC1 | AmpR, ScCYC1 terminator flanked with BsaI and predesigned 4-bp overhangs (7 and 8) | This work |
| pUC-78-YlCYC1 | AmpR, YlCYC1 terminator flanked with BsaI and predesigned 4-bp overhangs (7 and 8) | This work |
| pUC-78-POT1 | AmpR, POT1 terminator flanked with BsaI and predesigned 4-bp overhangs (7 and 8) | This work |
| pUC-78-Aco3 | AmpR, Aco3 terminator flanked with BsaI and predesigned 4-bp overhangs (7 and 8) | This work |
| pUC-78-XPR2 | AmpR, XPR2 terminator flanked with BsaI and predesigned 4-bp overhangs (7 and 8) | This work |
| pUC-89-GPD | AmpR, GPD promoter flanked with BsaI and predesigned 4-bp overhangs (8 and 9) | This work |
| pUC-89-GPDin | AmpR, GPDin promoter flanked with BsaI and predesigned 4-bp overhangs (8 and 9) | This work |
| pUC-89-EXP | AmpR, EXP promoter flanked with BsaI and predesigned 4-bp overhangs (8 and 9) | This work |
| pUC-89-TEF | AmpR, TEF promoter flanked with BsaI and predesigned 4-bp overhangs (8 and 9) | This work |
| pUC-89-TEFin | AmpR, TEFin promoter flanked with BsaI and predesigned 4-bp overhangs (8 and 9) | This work |
| pUC-89-ILV5 | AmpR, ILV5 promoter flanked with BsaI and predesigned 4-bp overhangs (8 and 9) | This work |
| pUC-89-FBAin | AmpR, FBAin promoter flanked with BsaI and predesigned 4-bp overhangs (8 and 9) | This work |
| pUC-89-YAT1 | AmpR, YAT1 promoter flanked with BsaI and predesigned 4-bp overhangs (8 and 9) | This work |
| pUC-910-ERG8 | AmpR, ERG8 gene flanked with BsaI and predesigned 4-bp overhangs (9 and 10) | This work |
| pUC-910-ERG19 | AmpR, ERG19 gene flanked with BsaI and predesigned 4-bp overhangs (9 and 10) | This work |
| pUC-910-IDI | AmpR, IDI gene flanked with BsaI and predesigned 4-bp overhangs (9 and 10) | This work |
| pUC-910-MrBBS | AmpR, MrBBS gene flanked with BsaI and predesigned 4-bp overhangs (9 and 10) | This work |
| pUC-1011-Mig1 | AmpR, Mig1 terminator flanked with BsaI and predesigned 4-bp overhangs (10 and 11) | This work |
| pUC-1011-Lip1 | AmpR, Lip1 terminator flanked with BsaI and predesigned 4-bp overhangs (10 and 11) | This work |
| pUC-1011-Lip2 | AmpR, Lip2 terminator flanked with BsaI and predesigned 4-bp overhangs (10 and 11) | This work |
| pUC-1011-ScCYC1 | AmpR, ScCYC1 terminator flanked with BsaI and predesigned 4-bp overhangs (10 and 11) | This work |
| pUC-1011-YlCYC1 | AmpR, YlCYC1 terminator flanked with BsaI and predesigned 4-bp overhangs (10 and 11) | This work |
| pUC-1011-POT1 | AmpR, POT1 terminator flanked with BsaI and predesigned 4-bp overhangs (10 and 11) | This work |
| pUC-1011-Aco3 | AmpR, Aco3 terminator flanked with BsaI and predesigned 4-bp overhangs (10 and 11) | This work |
| pUC-1011-XPR2 | AmpR, XPR2 terminator flanked with BsaI and predesigned 4-bp overhangs (10 and 11) | This work |
| pGGYL1-HT10X | KanR, HUH-P_TEF_-ERG10-T_XPR2_ | This work |
| pGGYL2-HT10X-G13M | KanR, HUH-P_TEF_-ERG10-T_XPR2_-P_GPD_-ERG13-T_Mig1_ | This work |
| pGGYL3-HT10X-G13M-EIL | KanR, HUH-P_TEF_-ERG10-T_XPR2_-P_GPD_-ERG13-T_Mig1_-P_EXP_-IDI-T_Lip2_ | This work |
| pGGYL1-HTBX | KanR, HUH-P_TEF_-MrBBS-T_XPR2_ | This work |
| pGGYL3-HTitY-Gi12S-G19L | KanR, HUH-P_TEF_-tHMG1-T_YlCYC1_-P_GPDin_-ERG12-T_ScCYC1_-P_GPD_-ERG19-T_Lip2_ | This work |
| pGGYL3-HT10X-G13M-GIL | KanR, HUH-P_TEF_-ERG10-T_XPR2_-P_GPD_-ERG13-T_Mig1_-P_GPD_-IDI-T_Lip2_ | This work |
| pGGYL2-HG8M-T20Y | KanR, HUH-P_GPD_-ERG8-T_Mig1_-P_TEF_-ERG20-T_YlCYC1_ | This work |
| pGGYL1-HTitX | KanR, HUH-P_TEFin_-tHMG1-T_XPR2_ | This work |
| pGGYL2-HTitX-EtL | KanR, HUH-P_TEFin_-tHMG1-T_XPR2_-P_EXP_-tHMG1-T_Lip1_ | This work |
| pGGYL1-HTiBS | KanR, HUH-P_TEFin_-MrBBS-T_XPR2_ | This work |
| pGGYL2-HTiBS-TBY | KanR, HUH-P_TEFin_-MrBBS-T_XPR2_-P_TEF_-MrBBS-T_CYC_ | This work |

**Table S2 Sequences of blocks and backbone plasmids used in this study**

| Names | DNA sequences (5’ to 3’) |
| --- | --- |
| HUH | ccggatcttccagtggtgcatgaacgcatgagaaagcccccggaagatcatcttccgggggctttttttttggcgcgcgatacagaccggttcagacaggaattctaccggggtgtgttctgtggagcattctcacttttggtaaacgacattgcttcaagtgcagcggaatcaaaaagtataaagtgggcagcgagtatacctgtacagactgtaggcgataactcaatccaattaccccccacaacatgactggccaaactgatctcaagactttattgaaatcagcaacaccgattctcaatgaaggcacatacttcttctgcaacattcacttgacgcctaaagttggtgagaaatggaccgacaagacatattctgctatccacggactgttgcctgtgtcggtggctacaatacgtgagtcagaagggctgacggtggtggttcccaaggaaaaggtcgacgagtatctgtctgactcgtcattgccgcctttggagtacgactccaactatgagtgtgcttggatcactttgacgatacattcttcgttggaggctgtgggtctgacagctgcgttttcggcgcggttggccgacaacaatatcagctgcaacgtcattgctggctttcatcatgatcacatttttgtcggcaaaggcgacgcccagagagccattgacgttctttctaatttggaccgatagccgtatagtccagtctatctataagttcaactaactcgtaactattaccataacatatacttcactgccccagataaggttccgataaaaagttctgcagactaaatttatttcagtctcctcttcaccaccaaaatgccctcctacgaagctcgagctaacgtccacaagtccgcctttgccgctcgagtgctcaagctcgtggcagccaagaaaaccaacctgtgtgcttctctggatgttaccaccaccaaggagctcattgagcttgccgataaggtcggaccttatgtgtgcatgatcaagacccatatcgacatcattgacgacttcacctacgccggcactgtgctccccctcaaggaacttgctcttaagcacggtttcttcctgttcgaggacagaaagttcgcagatattggcaacactgtcaagcaccagtacaagaacggtgtctaccgaatcgccgagtggtccgatatcaccaacgcccacggtgtacccggaaccggaatcattgctggcctgcgagctggtgccgaggaaactgtctctgaacagaagaaggaggacgtctctgactacgagaactcccagtacaaggagttcctggtcccctctcccaacgagaagctggccagaggtctgctcatgctggccgagctgtcttgcaagggctctctggccactggcgagtactccaagcagaccattgagcttgcccgatccgaccccgagtttgtggttggcttcattgcccagaaccgacctaagggcgactctgaggactggcttattctgacccccggggtgggtcttgacgacaagggagacgctctcggacagcagtaccgaactgttgaggatgtcatgtctaccggaacggatatcataattgtcggccgaggtctgtacggccagaaccgagatcctattgaggaggccaagcgataccagaaggctggctgggaggcttaccagaagattaactgttagaggttagactatggatatgtaatttaactgtgtatatagagagcgtgcaagtatggagcgcttgttcagcttgtatgatggtcagacgacctgtctgatcgagtatgtatgatactgcacaacctgtgtatccgcatgatctgtccaatggggcatgttgttgtgtttctcgatacggagatgctgggtacaagtagctaatacgattgaactacttatacttatatgaggcttgaagaaagctgacttgtgtatgacttattctcaactacatccccagtcacaataccaccactgcactaccactacaccaagctccggatcttccagtggtgcatgaacgcatgagaaagcccccggaagatcatcttccgggggctttttttttggcgcgcgatacagaccggttcagacagg |
| LEU2 | atggaacccgaaactaagaagaccaagactgactccaagaagattgttcttctcggcggcgacttctgtggccccgaggtgattgccgaggccgtcaaggtgctcaagtctgttgctgaggcctccggcaccgagtttgtgtttgaggaccgactcattggaggagctgccattgagaaggagggcgagcccatcaccgacgctactctcgacatctgccgaaaggctgactctattatgctcggtgctgtcggaggcgctgccaacaccgtatggaccactcccgacggacgaaccgacgtgcgacccgagcagggtctactcaagctgcgaaaggacctgaacctgtacgccaacctgcgaccctgccagctgctgtcgcccaagctcgccgatctctcccccatccgaaacgttgagggcaccgacttcatcattgtccgagagctcgtcggaggtatctactttggagagcgaaaggaggatgacggatctggcgtcgcttccgacaccgaaacctactccgttcctgaggttgagcgaattgcccgaatggccgccttcctggcccttcagcacaacccccctcttcccgtgtggtcccttgacaaggccaacgtgctggcctcctctcgactttggcgaaagactgtcactcgagtcctcaaggacgaattcccccagctcgagctcaaccaccagctgatcgactcggccgccatgatcctcatcaagcagccctccaagatgaatggtatcatcatcaccaccaacatgtttggcgatatcatctccgacgaggcctccgtcatccccggttctctgggtctgctgccctccgcctctctggcttctctgcccgacaccaacgaggcgttcggtctgtacgagccctgtcacggatctgcccccgatctcggcaagcagaaggtcaaccccattgccaccattctgtctgccgccatgatgctcaagttctctcttaacatgaagcccgccggtgacgctgttgaggctgccgtcaaggagtccgtcgaggctggtatcactaccgccgatatcggaggctcttcctccacctccgaggtcggagactttgttgccaacaaggtcaaggagctgctcaagaaggagtaagtcgtttctacgacgcattgatggaaggagcaaactgacgcgcctgcgggttggtctaccggcagggtccgctagtgtataa |
| URA3 | taccggggtgtgttctgtggagcattctcacttttggtaaacgacattgcttcaagtgcagcggaatcaaaaagtataaagtgggcagcgagtatacctgtacagactgtaggcgataactcaatccaattaccccccacaacatgactggccaaactgatctcaagactttattgaaatcagcaacaccgattctcaatgaaggcacatacttcttctgcaacattcacttgacgcctaaagttggtgagaaatggaccgacaagacatattctgctatccacggactgttgcctgtgtcggtggctacaatacgtgagtcagaagggctgacggtggtggttcccaaggaaaaggtcgacgagtatctgtctgactcgtcattgccgcctttggagtacgactccaactatgagtgtgcttggatcactttgacgatacattcttcgttggaggctgtgggtctgacagctgcgttttcggcgcggttggccgacaacaatatcagctgcaacgtcattgctggctttcatcatgatcacatttttgtcggcaaaggcgacgcccagagagccattgacgttctttctaatttggaccgatagccgtatagtccagtctatctataagttcaactaactcgtaactattaccataacatatacttcactgccccagataaggttccgataaaaagttctgcagactaaatttatttcagtctcctcttcaccaccaaaatgccctcctacgaagctcgagctaacgtccacaagtccgcctttgccgctcgagtgctcaagctcgtggcagccaagaaaaccaacctgtgtgcttctctggatgttaccaccaccaaggagctcattgagcttgccgataaggtcggaccttatgtgtgcatgatcaagacccatatcgacatcattgacgacttcacctacgccggcactgtgctccccctcaaggaacttgctcttaagcacggtttcttcctgttcgaggacagaaagttcgcagatattggcaacactgtcaagcaccagtacaagaacggtgtctaccgaatcgccgagtggtccgatatcaccaacgcccacggtgtacccggaaccggaatcattgctggcctgcgagctggtgccgaggaaactgtctctgaacagaagaaggaggacgtctctgactacgagaactcccagtacaaggagttcctggtcccctctcccaacgagaagctggccagaggtctgctcatgctggccgagctgtcttgcaagggctctctggccactggcgagtactccaagcagaccattgagcttgcccgatccgaccccgagtttgtggttggcttcattgcccagaaccgacctaagggcgactctgaggactggcttattctgacccccggggtgggtcttgacgacaagggagacgctctcggacagcagtaccgaactgttgaggatgtcatgtctaccggaacggatatcataattgtcggccgaggtctgtacggccagaaccgagatcctattgaggaggccaagcgataccagaaggctggctgggaggcttaccagaagattaactgttagaggttagactatggatatgtaatttaactgtgtatatagagagcgtgcaagtatggagcgcttgttcagcttgtatgatggtcagacgacctgtctgatcgagtatgtatgatactgcacaacctgtgtatccgcatgatctgtccaatggggcatgttgttgtgtttctcgatacggagatgctgggtacaagtagctaatacgattgaactacttatacttatatgaggcttgaagaaagctgacttgtgtatgacttattctcaactacatccccagtcacaataccaccactgcactaccactacacc |
| DsdA | atggaaaacgctaaaatgaactcgctcatcgcccagtatccgttggtaaaggatctggttgctcttaaagaaaccacctggtttaatcctggcacgacctcattggctgaaggtttaccttatgttggcctgaccgaacaggatgttcaggacgcccatgcgcgcttatcccgttttgcaccctatctggcaaaagcatttcctgaaactgctgccactggggggattattgaatcagaactggttgccattccggctatgcaaaaacggctggaaaaggaatatcaacaaccgatcagcgggcaactgttactgaaaaaagatagccatttgcccatttccggctccataaaagcacgcggcgggatttatgaagtcctggcacacgcagaaaaactggctctggaagcggggttgctgacgcttgatgatgactacagcaaactgctttctccggagtttaaacagttctttagccaatacagcattgctgtgggctcaaccggaaatctggggttatcaatcggcattatgagcgcccgcattggctttaaggtgacagttcatatgtctgctgatgcccgggcatggaaaaaagcgaaactgcgcagccatggcgttacggtcgtggaatatgagcaagattatggtgttgccgtcgaggaaggacgtaaagcagcgcagtctgacccgaactgtttctttattgatgacgaaaattcccgcacgttgttccttgggtattccgtcgctggccagcgtcttaaagcgcaatttgcccagcaaggccgtatcgtcgatgctgataaccctctgtttgtctatctgccgtgtggtgttggcggtggtcctggtggcgtcgcattcgggcttaaactggcgtttggcgatcatgttcactgcttttttgccgaaccaacgcactccccttgtatgttgttaggcgtccatacaggattacacgatcagatttctgttcaggatattggtatcgacaaccttaccgcagcggatggccttgcagttggtcgcgcatcaggctttgtcgggcgggcaatggagcgtctgctggatggcttctatacccttagcgatcaaaccatgtatgacatgcttggctggctggcgcaggaagaaggtattcgtcttgaaccttcggcactggcgggtatggccggacctcagcgcgtgtgtgcatcagtaagttaccaacagatgcacggtttcagcgcagaacaactgcgtaataccactcatctggtgtgggcgacgggaggtggaatggtgccggaagaagagatgaatcaatatctggcaaaaggccgttaa |
| Hyg | atgaaaaagcctgaactcaccgcgacgtctgtcgagaagtttctgatcgaaaagttcgacagcgtctccgacctgatgcagctctcggagggcgaagaatctcgtgctttcagcttcgatgtaggagggcgtggatatgtcctgcgggtaaatagctgcgccgatggtttctacaaagatcgttatgtttatcggcactttgcatcggccgcgctcccgattccggaagtgcttgacattggggagttcagcgagagcctgacctattgcatctcccgccgtgcacagggtgtcacgttgcaagacctgcctgaaaccgaactgcccgctgttctccagccggtcgcggaggccatggatgcgattgctgcggccgatcttagccagacgagcgggttcggcccattcggaccgcaaggaatcggtcaatacactacatggcgtgatttcatatgcgcgattgctgatccccatgtgtatcactggcaaactgtgatggacgacaccgtcagtgcgtccgtcgcgcaggctctcgatgagctgatgctttgggccgaggactgccccgaagtccggcacctcgtgcatgcggatttcggctccaacaatgtcctgacggacaatggccgcataacagcggtcattgactggagcgaggcgatgttcggggattcccaatacgaggtcgccaacatcctcttctggaggccgtggttggcttgtatggagcagcagacgcgctacttcgagcggaggcatccggagcttgcaggatcgccgcgcctccgggcgtatatgctccgcattggtcttgaccaactctatcagagcttggttgacggcaatttcgatgatgcagcttgggcgcagggtcgatgcgacgcaatcgtccgatccggagccgggactgtcgggcgtacacaaatcgcccgcagaagcgcggccgtctggaccgatggctgtgtagaagtactcgccgatagtggaaaccgacgccccagcactcgtccgagggcaaaggaatag |
| Neo | atgattgaacaagatggattgcacgcaggttctccggccgcttgggtggagaggctattcggctatgactgggcacaacagacaatcggctgctctgatgccgccgtgttccggctgtcagcgcaggggcgcccggttctttttgtcaagaccgacctgtccggtgccctgaatgaactgcaggacgaggcagcgcggctatcgtggctggccacgacgggcgttccttgcgcagctgtgctcgacgttgtcactgaagcgggaagggactggctgctattgggcgaagtgccggggcaggatctcctgtcatctcaccttgctcctgccgagaaagtatccatcatggctgatgcaatgcggcggctgcatacgcttgatccggctacctgcccattcgaccaccaagcgaaacatcgcatcgagcgagcacgtactcggatggaagccggtcttgtcgatcaggatgatctggacgaagagcatcaggggctcgcgccagccgaactgttcgccaggctcaaggcgcgcatgcccgacggcgaggatctcgtcgtgacccatggcgatgcctgcttgccgaatatcatggtggaaaatggccgcttttctggattcatcgactgtggccggctgggtgtggcggaccgctatcaggacatagcgttggctacccgtgatattgctgaagagcttggcggcgaatgggctgaccgcttcctcgtgctttacggtatcgccgctcccgattcgcagcgcatcgccttctatcgccttcttgacgagttcttctga |
| GPD | gacgcagtaggatgtcctgcacgggtctttttgtggggtgtggagaaaggggtgcttggagatggaagccggtagaaccgggctgcttggggggatttggggccgctgggctccaaagaggggtaggcatttcgttggggttacgtaattgcggcatttgggtcctgcgcgcatgtcccattggtcagaattagtccggataggagacttatcagccaatcacagcgccggatccacctgtaggttgggttgggtgggagcacccctccacagagtagagtcaaacagcagcagcaacatgatagttgggggtgtgcgtgttaaaggaaaaaaaaagaagcttgggttatattcccgctctatttagaggttgcgggatagacgccgacggagggcaatggcgccatggaaccttgcggatatcgatacgccgcggcggactgcgtccgaaccagctccagcagcgttttttccgggccattgagccgactgcgaccccgccaacgtgtcttggcccacgcactcatgtcatgttggtgttgggaggccactttttaagtagcacaaggcacctagctcgcagcaaggtgtccgaaccaaagaagcggctgcagtggtgcaaacggggcggaaacggcgggaaaaagccacgggggcacgaattgaggcacgccctcgaatttgagacgagtcacggccccattcgcccgcgcaatggctcgccaacgcccggtcttttgcaccacatcaggttaccccaagccaaacctttgtgttaaaaagcttaacatattataccgaacgtaggtttgggcgggcttgctccgtctgtccaaggcaacatttatataagggtctgcatcgccggctcaattgaatcttttttcttcttctcttctctatattcattcttgaattaaacacacatcaaca |
| GPDin | gacgcagtaggatgtcctgcacgggtctttttgtggggtgtggagaaaggggtgcttggagatggaagccggtagaaccgggctgcttggggggatttggggccgctgggctccaaagaggggtaggcatttcgttggggttacgtaattgcggcatttgggtcctgcgcgcatgtcccattggtcagaattagtccggataggagacttatcagccaatcacagcgccggatccacctgtaggttgggttgggtgggagcacccctccacagagtagagtcaaacagcagcagcaacatgatagttgggggtgtgcgtgttaaaggaaaaaaaaagaagcttgggttatattcccgctctatttagaggttgcgggatagacgccgacggagggcaatggcgccatggaaccttgcggatatcgatacgccgcggcggactgcgtccgaaccagctccagcagcgttttttccgggccattgagccgactgcgaccccgccaacgtgtcttggcccacgcactcatgtcatgttggtgttgggaggccactttttaagtagcacaaggcacctagctcgcagcaaggtgtccgaaccaaagaagcggctgcagtggtgcaaacggggcggaaacggcgggaaaaagccacgggggcacgaattgaggcacgccctcgaatttgagacgagtcacggccccattcgcccgcgcaatggctcgccaacgcccggtcttttgcaccacatcaggttaccccaagccaaacctttgtgttaaaaagcttaacatattataccgaacgtaggtttgggcgggcttgctccgtctgtccaaggcaacatttatataagggtctgcatcgccggctcaattgaatcttttttcttcttctcttctctatattcattcttgaattaaacacacatcaacaatggccatcaaagtcggtattaacggattcggacgaatcggacgaattgtgagtaccatagaaggtgatggaaacatgacccaacagaaacagatgacaagtgtcgtcgacccaccagagcccaattgagctcatactaacagtcgacaacctgtcgaaccaattgatgactccccgacaatgtactaacacaggtc |
| EXP | ggtaggtagacaatttacttttgcaaatgggcaacggcaaaaagcgccgggtgacctcgtggggtgttagtgtgggactgtaggaggtatatataaggagtttggcgcccgttttttcgagccccacacgtttcggtgagtatgagcggcggcagattcgagcgtttccggtttccgcggcgggacgagagcccatgatgggggctcccaccaccagcaatcagggccctgattacacacccacctgtaatgtcatgctgttcatcgtggttaatgctgctgtgtgctgtgtgtgtgtgttgtttggcgctcattgttgcgttatgcagcgtacaccacaatattggaagcttattagcctttctattttttcgtttgcaaggcttaacaacattgctgtggagagggatggggatatggaggccgctggagggagtcggagaggcgttttggagcggcttggcctggcgcccactcgcgaaacgcacctaggaccctttggcacgccgaaatgtgccacttttcagtctagtaacgccttacctacgtcattccatgcatgcatgtttgcgccttttttcccttgcccttgatcgccacacagtacagtgcactgtacagtggaggttttgggggggtcttagatgggagctaaaagcggcctagcggtacactagtgggattgtatggagtggcatggagcctgggtggagcctgacaggacgcacgaccggctagcccgtgacagacgatgggtggctcctgttgtccaccgcgtacaaatgtttgggccaaagtcttgtcagccttgcttgcgaacctaattcccaattttgtcacttcgcacccccattgatcgagccctaacccctgcccatcaggcaatccaattaagctcgcattgtctgccttgtttagtttggctcctgcccgtttcggcgtccacttgcacaaacacaaacaagcattatatataaggctcgtctctccctcccaaccacactcacttttttgcccgtcttcccttgctaacacaaaagtcaagaacacaaacaaccaccccaacccccttacacacaagacatatctacagca |
| TEF | acagaccgggttggcggcgtatttgtgtcccaaaaaacagccccaattgccccaattgaccccaaattgacccagtagcgggcccaaccccggcgagagcccccttcaccccacatatcaaacctcccccggttcccacacttgccgttaagggcgtagggtactgcagtctggaatctacgcttgttcagactttgtactagtttctttgtctggccatccgggtaacccatgccggacgcaaaatagactactgaaaatttttttgctttgtggttgggactttagccaagggtataaaagaccaccgtccccgaattacctttcctcttcttttctctctctccttgtcaactcacacccgaaatcgttaagcatttccttctgagtataagaatcattcaaa |
| TEFin | acagaccgggttggcggcgtatttgtgtcccaaaaaacagccccaattgccccaattgaccccaaattgacccagtagcgggcccaaccccggcgagagcccccttcaccccacatatcaaacctcccccggttcccacacttgccgttaagggcgtagggtactgcagtctggaatctacgcttgttcagactttgtactagtttctttgtctggccatccgggtaacccatgccggacgcaaaatagactactgaaaatttttttgctttgtggttgggactttagccaagggtataaaagaccaccgtccccgaattacctttcctcttcttttctctctctccttgtcaactcacacccgaaatcgttaagcatttccttctgagtataagaatcattcaaaatggtgagtttcagaggcagcagcaattgccacgggctttgagcacacggccgggtgtggtcccattcccatcgacacaagacgccacgtcatccgaccagcactttttgcagtactaaccgcag |
| ILV5 | aggagcgggagcggagttgattggagtggtgacaatgtcgctgtcgagaagcgatatcggtagctttcggagtgtcgatatcaccgagcctcagggaacaatggaggtccgggggacgttgaagtacagtatacctactgactgtcatatagtacgggatccagggattacgacagtgggtaggtctataagaaacagaggctcatcagctatggggaaagattaacaggtacagttgtatgaaacttgaaagtggcagcacagacactcttatactacgactacttgtagtactgtactgggagtctcctccttgaagcctgtacgaaagtcgcctacaagactgtccccaagtccggatctgttggactctggcatccatcttggcccagatcggatctaagacactggcgtgtcgctaaactaaccctccgacggggctgttcattcacctccggtctattaaaagtgtaataatggtctttgttagtctgtctggaggcgagagaattgtggggtaagatgatggagtgggtgtagtgagtttagtgagagagtgtggtagcgagtggacggctagatgtagagctcgaattgagtggttatgagtggaccatgttgtactccggccgagattacaagtggttgaacgccatcaacctcatcaacgacgtcgctgaaaaatgacggaatcttcaactcttggatgtattaacaatcccgtgacaatcagacaccagccaactccagcagacagagcaattgatctaaccatactactgtacctgtagtacgtaatacagtagtaccagatgtacagtgagtcaccaccacctatcttgtaccccatctccacccagctccatgactcaatgcttctgtctcaccgctcgtcaccgctcgtcaccgctcgtcaccgctgctgcctcgccgttcccatcggcccctacatcggcatccacatcggcatccacatcggcatcaccatcggcatatcactcactcactgtctgcatctatccatgactcttttttcgctgcaaaattcaaaccgtaaatccactccagcgacatcggcagactgctgccaaaccttgatctgatttgcggcctcccgattggccaattgccaacctaaacttttcatctgctcccaatcgccttgttgtcggagcactgtaaccttagctccaaagtgcccaacgtggtctatataacgacgctgaaagtccgccgattttttctctctttcttctctcacaaccaatcccccccaacacaacaccacagta |
| FBAin | gtacgtagcaacaacagtgtacgcagtactatagaggaacaattgccccggagaagacggccaggccgcctagatgacaaattcaacaactcacagctgactttctgccattgccactaggggggggcctttttatatggccaagccaagctctccacgtcggttgggctgcacccaacaataaatgggtagggttgcaccaacaaagggatgggatggggggtagaagatacgaggataacggggctcaatggcacaaataagaacgaatactgccattaagactcgtgatccagcgactgacaccattgcatcatctaagggcctcaaaactacctcggaactgctgcgctgatctggacaccacagaggttccgagcactttaggttgcaccaaatgtcccaccaggtgcaggcagaaaacgctggaacagcgtgtacagtttgtcttagcaaaaagtgaaggcgctgaggtcgagcagggtggtgtgacttgttatagcctttagagctgcgaaagcgcgtatggatttggctcatcaggccagattgagggtctgtggacacatgtcatgttagtgtacttcaatcgccccctggatatagccccgacaataggccgtggcctcatttttttgccttccgcacatttccattgctcggtacccacaccttgcttctcctgcacttgccaaccttaatactggtttacattgaccaacatcttacaagcggggggcttgtctagggtatatataaacagtggctctcccaatcggttgccagtctcttttttcctttctttccccacagattcgaaatctaaactacacatcacacaatgcctgttactgacgtccttaagcgaaagtccggtgtcatcgtcggcgacgatgtccgagccgtgagtatccacgacaagatcagtgtcgagacgacgcgttttgtgtaatgacacaatccgaaagtcgctagcaacacacactctctacacaaactaacccagctcttc |
| YAT1 | acatggtttttagggggtactgtacatatatatctgtggtggtcctgattttcgccaaaccatgttcttcgtgttccttttcaccctcactcacatgtcgtccacttgttagcgtcatctttcttggcaatagctactattcaacattgaaggaacagccgtcccgaaagtcacttgtcggagtactccgtcccgcgacgcatgcaaccgctatgaactaacccgtgtcagtgaagtcgggggatagtcttctgccattgttggtgaaactgttctgcttattcacgtgaatttcaacattcacacatcgatcgacaagcgaggctattatttaaaaactgtcttagtgagttaccctgctgacgaagcaatgataatctgattcgaggagagatgatgaacacgactgaagtgagtggttattcccccattaacgataatatccgcattaattattatgctgcacggaactgacatcaatatcgcccaccaccactcgtttcaacacgttgacaggctgatgacaccagctagacgccaaattttagtgatctaaacatcctcttttggtaagggtaggtttcagagcggggctagcgggaatgccaagcgttagctggggtatgtgagcattgcgccaaaaaacgactgtacgagatggaccaagacggggccaagctgtgcagaaagtgaagaacaagcagcagccgattttagaaaggagcgacagaacgcttaagagagactcgcgtgactaaacagccttctactccgctttcatcacccaagtaagtatgttgacagccactcaccttcaccggtttgcgtttgacaatacagttggacccctgcagagatactacccacgtgggtggtatcgagctgtaattggcatccttcagtaataaacctgaccagccgtcagtcgccgagaaccaagaacacgggctagccaatcacgcagatccaattagccatcaaagtctttgttattgagtctccacaacgattaaccagtctaaacaagcatacgaatacgagctttctcttgaaaagaatggaaagaaaacatatatagactgtgggacagacgaggcaggagaaaaggaaccttaccccaagtcgctcgttcaaca |
| ERG10 | atgcgactcactctgccccgacttaacgccgcctacattgtaggagccgcccgaactcctgtcggcaagttcaacggagccctcaagtccgtgtctgccattgacctcggtatcaccgctgccaaggccgctgtccagcgatccaaggtccccgccgaccagattgacgagtttctgtttggccaggtgctgaccgccaactccggccaggcccccgcccgacaggtggttatcaagggtggtttccccgagtccgtcgaggccaccaccatcaacaaggtgtgctcttccggcctcaagaccgtggctctggctgcccaggccatcaaggccggcgaccgaaacgttatcgtggccggtggaatggagtccatgtccaacaccccctactactccggtcgaggtcttgttttcggcaaccagaagctcgaggactccatcgtcaaggacgggctctgggacccctacaacaacatccacatgggcaactgctgcgagaacaccaacaagcgagacggcatcacccgagagcagcaggacgagtacgccatcgagtcctaccgacgggccaacgagtccatcaagaacggcgccttcaaggacgagattgtccccgttgagatcaagacccgaaagggcaccgtgactgtctccgaggacgaggagcccaagggagccaacgccgagaagctcaagggcctcaagcctgtctttgacaagcagggctccgtcactgccggtaacgcctcccccatcaacgatggtgcttctgccgttgtcgttgcctctggcaccaaggccaaggagctcggtacccccgtgctcgccaagattgtctcttacgcagacgccgccaccgcccccattgactttaccattgctccctctctggccattcccgccgccctcaagaaggctggccttaccaaggacgacattgccctctgggagatcaacgaggccttctccggtgtcgctctcgccaacctcatgcgactcggaattgacaagtccaaggtcaacgtcaagggtggagctgttgctctcggccaccccattggtgcctccggtaaccgaatctttgtgactttggtcaacgccctcaaggagggcgagtacggagttgccgccatctgcaacggtggaggagcttccaccgccatcgtcatcaagaaggtttcttctgtcgagtag |
| tHMG1 | atgacccagtctgtgaaggtggttgagaagcacgttcctatcgtcattgagaagcccagcgagaaggaggaggacacctcttctgaagactccattgagctgactgtcggaaagcagcccaagcccgtgaccgagacacgttctctggacgacctagaggctatcatgaaggcaggtaagaccaagcttctggaggaccacgaggttgtcaagctctctctcgagggcaagcttcctttgtatgctcttgagaagcagcttggtgacaacacccgagctgttggcatccgacgatctatcatctcccagcagtctaataccaagactttagagacatcaaagcttccttacctgcactacgactacgaccgtgtttttggagcctgttgcgagaacgttattggttacatgcctctccccgttggtgttgctggccccatgaacattgatggcaagaactaccacattcctatggccaccactgagggttgtcttgttgcctcaaccatgcgaggttgcaaggccatcaacgccggtggcggtgttaccactgtgcttactcaggacggtatgacacgaggtccttgtgtttccttcccctctctcaagcgggctggagccgctaagatctggcttgattccgaggaggggctcaagtccatgcgaaaggccttcaactccacctctcgatttgctcgtctccagtctcttcactctacccttgctggtaacctgctgtttattcgattccgaaccaccactggtgatgccatgggcatgaacatgatctccaagggcgtcgaacactctctggccgtcatggtcaaggagtacggcttccctgatatggacattgtgtctgtctcgggtaactactgcactgacaagaagcccgcagcgatcaactggatcgaaggccgaggcaagagtgttgttgccgaagccaccatccctgctcacattgtcaagtctgttctcaaaagtgaggttgacgctcttgttgagctcaacatcagcaagaatctgatcggtagtgccatggctggctctgtgggaggtttcaatgcacacgccgcaaacctggtgaccgccatctaccttgccactggccaggatcctgctcagaatgtcgagtcttccaactgcatcacgctgatgagcaacgtcgacggtaacctgctcatctccgtttccatgccttctatcgaggtcggtaccattggtggaggtactattttggagccccagggggctatgctggagatgcttggcgtgcgaggtcctcacatcgagacacccggtgccaacgcccaacagcttgctcgcatcattgcttctggagttcttgcagcggagctttcgctgtgttctgctcttgctgccggccatcttgtgcaaagtcatatgacccacaaccggtcccaggctcctactccggccaagcagtctcaggccgatctgcagcgtctacaaaacggttcgaatatttgcatacggtcatag |
| ERG13 | atgtcgcaaccccagaacgttggaatcaaagccctcgagatctacgtgccttctcgaattgtcaaccaggctgagctcgagaagcacgacggtgtcgctgctggcaagtacaccattggtcttggtcagaccaacatggcctttgtcgacgacagagaggacatctattcctttgccctgaccgccgtctctcgactgctcaagaacaacaacatcgaccctgcatctattggtcgaatcgaggttggtactgaaacccttctggacaagtccaagtccgtcaagtctgtgctcatgcagctctttggcgagaacagcaacattgagggtgtggacaacgtcaacgcctgctacggaggaaccaacgccctgttcaacgctatcaactgggttgagggtcgatcttgggacggccgaaacgccatcgtcgttgccggtgacattgccctctacgcaaagggcgctgcccgacccaccggaggtgccggctgtgttgccatgctcattggccccgacgctcccctggttcttgacaacgtccacggatcttacttcgagcatgcctacgatttctacaagcctgatctgacctccgagtacccctatgttgatggccactactccctgacctgttacacaaaggccctcgacaaggcctacgctgcctacaacgcccgagccgagaaggtcggtctgttcaaggactccgacaagaagggtgctgaccgatttgactactctgccttccacgtgcccacctgcaagcttgtcaccaagtcttacgctcgacttctctacaacgactacctcaacgacaagagcctgtacgagggccaggtccccgaggaggttgctgccgtctcctacgatgcctctctcaccgacaagaccgtcgagaagaccttccttggtattgccaaggctcagtccgccgagcgaatggctccttctctccagggacccaccaacaccggtaacatgtacaccgcctctgtgtacgcttctctcatctctctgctgacttttgtccccgctgagcagctgcagggcaagcgaatctctctcttctcttacggatctggtcttgcttccactcttttctctctgaccgtcaagggagacatttctcccatcgtcaaggcctgcgacttcaaggctaagctcgatgaccgatccaccgagactcccgtcgactacgaggctgccaccgatctccgagagaaggcccacctcaagaagaactttgagccccagggagacatcaagcacatcaagtctggcgtctactacctcaccaacatcgatgacatgttccgacgaaagtacgagatcaagcagtag |
| ERG12 | atggactacatcatttcggcgccaggcaaagtgattctatttggtgaacatgccgctgtgtttggtaagcctgcgattgcagcagccatcgacttgcgaacatacctgcttgtcgaaaccacaacatccgacaccccgacagtcacgttggagtttccagacatccacttgaacttcaaggtccaggtggacaagctggcatctctcacagcccagaccaaggccgaccatctcaattggtcgactcccaaaactctggataagcacattttcgacagcttgtctagcttggcgcttctggaagaacctgggctcactaaggtccagcaggccgctgttgtgtcgttcttgtacctctacatccacctatgtcccccttctgtgtgcgaagattcatcaaactgggtagttcgatcaacgctgcctatcggcgcgggcctgggctcttccgcatccatttgtgtctgtttggctgcaggtcttctggttctcaacggccagctgagcattgaccaggcaagagatttcaagtccctgaccgagaagcagctgtctctggtggacgactggtccttcgtcggtgaaatgtgcattcacggcaacccgtcgggcatcgacaatgctgtggctactcagggaggtgctctgttgttccagcgacctaacaaccgagtccctcttgttgacattcccgagatgaagctgctgcttaccaatacgaagcatcctcgatctaccgcagacctggttggtggagtcggagttctcactaaagagtttggctccatcatggatcccatcatgacttcagtaggcgagatttccaaccaggccatggagatcatttctagaggcaagaagatggtggaccagtctaaccttgagattgagcagggtatcttgcctcaacccacctctgaggatgcctgcaacgtgatggaagatggagctactcttcaaaagttgagagatatcggttcggaaatgcagcatctagtgagaatcaatcacggcctgcttatcgctatgggtgtttcccacccgaagctcgaaatcattcgaactgcctccattgtccacaacctgggtgagacaaagctcactggtgctggaggaggaggttgcgccatcactctagtcacttctaaagacaagactgcgacccagctggaggaaaatgtcattgctttcacagaggagatggctacccatggcttcgaggtgcacgagactactattggtgccagaggagttggtatgtgcattgatcatccctctctcaagactgttgaagccttcaagaaggtggagcgggcggatctcaaaaacatcggtccctggacccattag |
| ERG8 | atgaccacctattcggctccgggaaaggccctcctttgcggcggttatttggttattgatccggcgtattcagcatacgtcgtgggcctctcggcgcgtatttacgcgacagtttcggcttccgaggcctccaccacctctgtccatgtcgtctctccgcagtttgacaagggtgaatggacctacaactacacgaacggccagctgacggccatcggacacaacccatttgctcacgcggccgtcaacaccgttctgcattacgttcctcctcgaaacctccacatcaacatcagcatcaaaagtgacaacgcgtaccactcgcaaattgacagcacgcagagaggccagtttgcataccacaaaaaggcgatccacgaggtgcctaaaacgggcctcggtagctccgctgctcttaccaccgttcttgtggcagctttgctcaagtcatacggcattgatcccttgcataacacccacctcgttcacaacctgtcccaggttgcacactgctcggcacagaagaagattgggtctggatttgacgtggcttcggccgtttgtggctctctagtctatagacgtttcccggcggagtccgtgaacatggtcattgcagctgaagggacctccgaatacggggctctgttgagaactaccgttaatcaaaagtggaaggtgactctggaaccatccttcttgccgccgggaatcagcctgcttatgggagacgtccagggaggatctgagactccaggtatggtggccaaggtgatggcatggcgaaaagcaaagccccgagaagccgagatggtgtggagagatctcaacgctgccaacatgctcatggtcaagttgttcaacgacctgcgcaagctctctctcactaacaacgaggcctacgaacaacttttggccgaggctgctcctctcaacgctctaaaaatgataatgttgcagaaccctctcggagaactagcacgatgcattatcactattcgaaagcatctcaagaagatgacacgggagactggtgctgctattgagccggatgagcagtctgcattgctcaacaagtgcaacacttatagtggagtcattggaggtgttgtgcctggagcaggaggctacgatgctatttctcttctggtgatcagctctacggtgaacaatgtcaagcgagagagccagggagtccaatggatggagctcaaggaggagaacgagggtctgcggctcgagaaggggttcaagtag |
| ERG19 | atgatccaccaggcctccaccaccgctccggtgaacattgcgacactcaagtactggggcaagcgagatcctgctctcaatctgcccactaacaactccatctccgtgactttgtcgcaggatgatctgcggaccctcaccacagcctcgtgttcccctgatttcacccaggacgagctgtggctcaatggcaagcaggaggacgtgagcggcaaacgtctggttgcgtgtttccgagagctgcgggctctgcgacacaaaatggaggactccgactcttctctgcctaagctggccgatcagaagctcaagatcgtgtccgagaacaacttccccaccgccgctgggctcgcctcatcggctgctggctttgccgccctgatccgagccgttgcaaatctctacgagctccaggagacacccgagcagctgtccattgtggctcgacagggctctggatccgcctgtcgatctctctacggaggctacgtggcatgggaaatgggcaccgagtctgacggaagcgactcgcgagcggtccagatcgccaccgccgaccactggcccgagatgcgagccgccatcctcgttgtctctgccgacaagaaggacacgtcgtccactaccggtatgcaggtgactgtgcacacttctcccctcttcaaggagcgagtcaccactgtggttcccgagcggtttgcccagatgaagaagtcgattctggaccgagacttccccacctttgccgagctcaccatgcgagactcaaaccagttccacgccacctgtctggactcgtatcctcccattttctacctcaacgacgtgtcgcgagcctccattcgggtagttgaggccatcaacaaggctgccggagccaccattgccgcctacacctttgatgctggacccaactgtgtcatctactacgaggacaagaacgaggagctggttctgggtgctctcaaggccattctgggccgtgtggagggatgggagaagcaccagtctgtggacgccaagaagattgatgttgacgagcggtgggagtccgagctggccaacggaattcagcgggtgatccttaccaaggttggaggagatcccgtgaagaccgctgagtcgcttatcaacgaggatggttctctgaagaacagcaagtag |
| IDI | atgacgacgtcttacagcgacaaaatcaagagtatcagcgtgagctctgtggctcagcagtttcctgaggtggcgccgattgcggacgtgtccaaggctagccggcccagcacggagtcgtcggactcgtcggccaagctatttgatggccacgacgaggagcagatcaagctgatggacgagatctgtgtggtgctggactgggacgacaagccgattggcggcgcgtccaaaaagtgctgtcatctgatggacaacatcaacgacggactggtacatcgggccttttccgtgttcatgttcaacgaccgcggtgagctgcttctgcagcagcgggcggcggaaaaaatcacctttgccaacatgtggaccaacacgtgctgctcgcatcctctggcggtgcccagcgagatgggcgggctggatctggagtcccggatccagggcgccaaaaacgccgcggtccggaagcttgagcacgagctgggaatcgaccccaaggccgttccggcagacaagttccatttcctcacccggatccactacgccgcgccctcctcgggcccctggggcgagcacgagattgactacattctgtttgtccggggcgaccccgagctcaaggtggtggccaacgaggtccgcgataccgtgtgggtgtcgcagcagggactcaaggacatgatggccgatcccaagctggttttcaccccttggttccggctcatttgtgagcaggcgctgtttccctggtgggaccagttggacaatctgcccgcgggcgatgacgagattcggcggtggatcaagtag |
| ERG20 | atgtccaaggcgaaattcgaaagcgtgttcccccgaatctccgaggagctggtgcagctgctgcgagacgagggtctgccccaggatgccgtgcagtggttttccgactcacttcagtacaactgtgtgggtggaaagctcaaccgaggcctgtctgtggtcgacacctaccagctactgaccggcaagaaggagctcgatgacgaggagtactaccgactcgcgctgctcggctggctgattgagctgctgcaggcgtttttcctcgtgtcggacgacattatggatgagtccaagacccgacgaggccagccctgctggtacctcaagcccaaggtcggcatgattgccatcaacgatgctttcatgctagagagtggcatctacattctgcttaagaagcatttccgacaggagaagtactacattgaccttgtcgagctgttccacgacatttcgttcaagaccgagctgggccagctggtggatcttctgactgcccccgaggatgaggttgatctcaaccggttctctctggacaagcactcctttattgtgcgatacaagactgcttactactccttctacctgcccgttgttctagccatgtacgtggccggcattaccaaccccaaggacctgcagcaggccatggatgtgctgatccctctcggagagtacttccaggtccaggacgactaccttgacaactttggggaccccgagttcattggtaagatcggcaccgacatccaggacaacaagtgctcctggctcgttaacaaagcccttcagaaggccacccccgagcagcgacagatcctcgaggacaactacggcgtcaaggacaagtccaaggagctcgtcatcaagaaactgtatgatgacatgaagattgagcaggactaccttgactacgaggaggaggttgttggcgacatcaagaagaagatcgagcaggttgacgagagccgaggcttcaagaaggaggtgctcaacgctttcctcgccaagatttacaagcgacagaagtag |
| MrBBS | atgtctaccctctccgtgtccactccttccttctcctcctcccccctctcttccgtgaacaaaaactccaccaagcagcacgtcacccgaaactccgtgatcttccatgactctatctggggcgaccagttcctcgaatataaagagaagtttaatgtcgctactgaaaagcagctgatcgaggaactcaaggaggaggtccgaaacgaactcatgatccgagcttgcaacgaggcctcccgatacattaaactcatccagctgatcgacgtcgtggaacgactgggcctcgcttatcacttcgagaaggagatcgaagagtccctccaacacatctacgtcacctacggccacaagtggaccaactacaacaacatcgagtctctctctctctggtttcgactcctccgacagaacggtttcaacgtgtcttccgatatctttgaaaaccatatcgacgaaaagggcaactttcaagaatctttatgcaacgacccccaaggtatgctggctttatacgaggccgcctacatgagagtcgagggtgagatcattttagataaggccctcgagtttaccaagctgcacctcggcatcatttctaacgacccctcttgcgattcctctctgcgaactgagatcaagcaagctttaaaacagcctctccgacgaagactgcctcgactggaggccgtccgatatatcgctatttaccagcagaaggcctcccactctgaggttttactcaagctcgctaaactggactttaacgtgctccaagaaatgcacaaggatgagctgtcccagatctgcaaatggtggaaggacctcgacatccgaaacaagctcccctacgtcagagatcgactgattgagggctacttctggattttaggcatctactttgagccccagcactcccgaacccgaatgtttttaatgaagacttgtatgtggctcatcgttttagacgacacctttgacaactacggcacttacgaggagctggagatcttcacccaagctgtcgagcgatggtccatcacttgtctggacgagctgcccgagtacatgaagctcatctaccatgagcagttccgagtccatcaagagatggaggagtccctcgagaaagagggcaaggcctaccagatccattacatcaaagagatggccaaggagggcactcgatccttattactggaggccaagtggctgaaggagggttatatgcccaccctcgacgagtacctctccaactctttagtcacttgtggctacgctctcatgactgccagatcttacgtcgctcgagacgatggtatcgtgaccgaggacgctttcaaatgggtcgctactcacccccctatcgtgaaggccgcttgtaagattctccgactcatggacgacatcgccacccataaggaggagcaagaacgaggccacatcgcctcttctatcgagtgctacagaaaggaaaccggcgcctctgaggaggaggcttgcatggactttctcaagcaagttgaagatggctggaaggtcattaaccaagaatctctcatgcctaccgacgtgcccttccccttattaattcccgctatcaacctcgcccgagtgtccgacactttatataaagacaacgacggctacaatcacgccgataaggaggtgatcggctacatcaagtccctcttcgtccaccctatgattgtgtaa |
| Mig1 | cactggccggtcgataatttaacgtgctgagctcagcacacgcattgcccattggctgtatatagatgaatgtaatgataccgtaagagaatgagagcacggtattgtattacaggggattaagtacacattacttggagttctgtaccagaagacactactatacatggtattacttacattagagtcggtgaccgtattcgtctcgtatagacataatattttcctaccccacattgttcctgggccttcggagcacatctacagtgagtgactgtttcagttgagcttgaggggttaagtaagtgggggaagggtttgcgattctgaaaaagagcatgactaatctctctgtggaggagcaatgaagtcacgtgatgcaatcataccggtgtatcggatctgcctgggtgtctgattactaatcatttactcacctgttttccccagctatctcatccatctcagagcctcggcccagccttcggcccttttgggttt |
| Lip1 | ggttcatgagaagataaatatataaatacattgagatattaaatgcgctagattagagagcctcatactgctcggagagaagccaagacgagtactcaaaggggattacaccatccatatccacagacacaagctggggaaaggttctatatacactttccggaataccgtagtttccgatgttatcaatgggggcagccaggatttcaggcacttcggtgtctcggggtgaaatggcgttcttggcctccatcaagtcgtaccatgtcttcatttgcctgtcaaagtaaaacagaagcagatgaagaatgaacttgaagtgaaggacgtgactacaacagcctgcctggtcaaccacatcattcatgttgctgatgatcttctgaatcagctgaagagggtactctgtctcaatcagatcaccaaatttggggtgcagtagggcacatgatcgaagcatgagtgcagtagtagcacaataagtcattcgagcaaggtagagagtactcgacgacagaatctgcattgtgtagagctccgagaatccctccagggatgttcgcaccgcatcggcagtggccatggcactattgatcactgcgggggtcagtttgagatcgaagatgttcagaccaaactcggagaagggcgttcgaggaacagtgggtgacacgttcgtcaacagagatagttcatgcagagagatcttgagagaagatttgaacaactcgagaacgtgatcaattgggaaccctcgcttgtcaaaggtgaacacctcgtcgattcgacggtcgaaatgcttgaccagccgctgagtacgttcatcattgatatccagaggcttcttgtaatcctcacttctaagcatggccatcatatcttccaact |
| Lip2 | gctatttatcactctttacaacttctacctcaactatctactttaataaatgaatatcgtttattctctatgattactgtatatgcgttcctctaagacaaatcgaaaccagcatgcgatcgaatggcatacaaaagtttcttccgaagttgatcaatgtcctgatagtcaggcagcttgagaagattgacacaggtggaggccgtagggaaccgatcaacctgtctaccagcgttacgaatggcaaatgacgggttcaaagccttgaatccttgcaatggtgccttggatactgatgtcacaaacttaagaagcagccgcttgtcctcttcctcgaaactctcaaacacagtccagaagtcctttatagtttgatctgtatccagatagcctccgtaattggtgtgtgtcttcaaatcccagacgtccacattggcatgtcctccactgataagcatttgaagttcatctgcgttgaacattgagac |
| ScCYC1 | tcatgtaattagttatgtcacgcttacattcacgccctccccccacatccgctctaaccgaaaaggaaggagttagacaacctgaagtctaggtccctatttatttttttatagttatgttagtattaagaacgttatttatatttcaaatttttcttttttttctgtacagacgcgtgtacgcatgtaacattatactgaaaaccttgcttgagaaggttttgggacgctcgaaggctttaatttgc |
| YlCYC1 | taagcgtctacaactggacccttagcctgtatatatcaattgattatttaaagatttggtcggtaggcggttcgtattgtacaatgggatctgttactgaggtggatctacccaacttgcgagattcaattgcgagattcaatcgcgagattcaattgcgagaatcagttgcgagttgttctaacactcagcttctacgagcgcttgtattaggacgagtgatactccgtggggcgacggcttctcttgcgtcttctgttgtattctttcttacactatcgtccatctccaaccacctcg |
| POT1 | atgtacatacaagattatttatagaaatgaatcgcgatcgaacaaagagtacgagtgtacgagtaggggatgatgataaaagtggaagaagttccgcatctttggatttatcaacgtgtaggacgatacttcctgtaaaaatgcaatgtctttaccataggttctgctgtagatacttgtacagttgcagaccagttggagtatagaatggtacacttaccaaaaagtgttgatggttgtaactacgatacgggatccccgctgatatgcctaaggaacaatcaaagaggaagatattaattcagaatgctagtatacagttagggatggtgagtgttgggtgatgcaggggaggttgtcgagcggttcacttgagtgcgtcccgctgtttcatcaagtgcatttcaagaatcccgttggccatgtgcattttgaccttttgcgggtcgattttgaccgttggcggaaaccgcaccgttctctggaaggagccgttgtgtcgttctcccaccatgagacagtcggacagatgtgcaaaggggttggtgtagtggccactgatggtcaggctgttggagtctgcgtcgtaggcggtttccaggtccgccgaccgcaccgccggcagacttgtcaccacaatgtacatgtttcccaggtcgtagatgtcgactggtggcgcgaatgggtcagtatgggagtgtgggagaggagtagctggtggcgagtgggccaggtctgtttcggcatagtcgggcattacaaaggacatggggagggcgacgggaggctttttgaggagttgaagaagagctggggaagaaatcaagtgtcaagctt |
| Aco3 | atggagcgtgtgttctgagtcgatgttttctatggagttgtgagtgttagtagacatgatgggtttatatatgatgaatgaatagatgtgattttgatttgcacgatggaattgagaactttgtaaacgtacatgggaatgtatgaatgtgggggttttgtgactggataactgacggtcagtggacgccgttgttcaaatatccaagagatgcgagaaactttgggtcaagtgaacatgtcctctctgttcaagtaaaccatcaactatgggtagtatatttagtaaggacaagagttgagattctttggagtcctagaaacgtattttcgcgttccaagatcaaattagtagagtaatacgggcacgggaatccattcatagtctcaattttcccataggtgtgctacaaggtgttgagatgtggtacagtaccaccatgattcgaggtaaagagcccagaagtcattgatgaggtcaagaaatacacagatctacagctcaatacaatgaatatcttctttcatattcttcaggtgacaccaagggtgtctattttccccagaaatgcgtgaaaaggcgcgtgtgtagcgtggagtatgggttcggttggcgtatccttcatatatcgacgaaatagtagggcaagagatgacaaaaagtatctatatgtagacagcgtagaatatggatttgattggtataaattcatttattgcgtgtctcacaaatactctcgataagttggggttaaactggagatggaacaatgtcgatatctcgacatattttgatatttgtactgttgatagtgataaaaagtagaccgttcgaatctcgacaaggagaagagtccaatgaaataggtttcatcatcatttgtcatagttaaacgccgctggttgccattactatccgtcttgactacaaccccaactcagcctagaccacagcgaagagaatcagtttggagactgaaaatgagctccaatcaatacatattagggtattaccaagat |
| XPR2 | gatccaactacggaacttgtgttgatgtctttgcccccggctccgatatcatctctgcctcttaccagtccgactctggtactttggtctactccggtacctccatggcctgtccccacgttgccggtcttgcctcctactacctgtccatcaatgacgaggttctcacccctgcccaggtcgaggctcttattactgagtccaacaccggtgttcttcccaccaccaacctcaagggctctcccaacgctgttgcctacaacggtgttggcatttaggcaattaacagatagtttgccggtgataattctcttaacctcccacactcctttgacataacgatttatgtaacgaaactgaaatttgaccagatattgttgtaaatagaaaatctggcttgtaggtggcaaaatcccgtctttgttcatcaattccctctgtgactactcgtcatccctttatgttcgactgtcgtatttttattttccatacatacgcaagtgagatgcccgtgtc |
| PGGYL1 | gcgcccaatacgcaaaccgcctctccccgcgcgttggccgattcattaatgcagctggcacgacaggtttcccgactggaaagcgggcagtgagcgcaacgcaattaatgtgagttagctcactcattaggcaccccaggctttacactttatgcttccggctcgtatgttgtgtggaattgtgagcggataacaatttcacacaggaaacagctATTTAAATctagggagaccatgaccatgattacgccaagcttgcatgcctgcaggtcgactctagaggatccccgggtaccgagctcgaattcactggccgtcgttttacaacgtcgtgactgggaaaaccctggcgttacccaacttaatcgccttgcagcacatccccctttcgccagctggcgtaatagcgaagaggcccgcaccgatcgcccttcccaacagttgcgcagcctgaatggcgaatggcgcctgatgcggtattttctccttacgcatctgtgcggtatttcacaccgcatatggtgcactctcagtacaatctgctctgatgccgcatagGGTCTCaGCCTATTTAAATttaagccagccccgacacccgccaacacccgctgacgcgccctgacgggcttgtctgctcccggcatccgcttacagacaagctgtgaccgtctccgggagctgcatgtgtcagaggttttcaccgtcatcaccgaaacgcgcgagacgaaagggcctcgtgatacgcctatttttataggttaatgtcatgataataatggtttcttagacgtcaggtggcacttttcggggaaatgtgcgcggaacccctatttgtttatttttctaaatacattcaaatatgtatccgctcatgagacaataaccctgataaatgcttcaataatattgaaaaaggaagagtatgagccatattcaacgggaaacgtcttgctctaggccgcgattaaattccaacatggatgctgatttatatgggtataaatgggctcgcgataatgtcgggcaatcaggtgcgacaatctatcgattgtatgggaagcccgatgcgccagagttgtttctgaaacatggcaaaggtagcgttgccaatgatgttacagatgagatggtcagactaaactggctgacggaatttatgcctcttccgaccatcaagcattttatccgtactcctgatgatgcatggttactcaccactgcgatccccgggaaaacagcattccaggtattagaagaatatcctgattcaggtgaaaatattgttgatgcgctggcagtgttcctgcgccggttgcattcgattcctgtttgtaattgtccttttaacagcgatcgcgtatttcgtctcgctcaggcgcaatcacgaatgaataacggtttggttgatgcgagtgattttgatgacgagcgtaatggctggcctgttgaacaagtctggaaagaaatgcataaacttttgccattctcaccggattcagtcgtcactcatggtgatttctcacttgataaccttatttttgacgaggggaaattaataggttgtattgatgttggacgagtcggaatcgcagaccgataccaggatcttgccatcctatggaactgcctcggtgagttttctccttcattacagaaacggctttttcaaaaatatggtattgataatcctgatatgaataaattgcagtttcatttgatgctcgatgagtttttctaactgtcagaccaagtttactcatatatactttagattgatttaaaacttcatttttaatttaaaaggatctaggtgaagatcctttttgataatctcatgaccaaaatcccttaacgtgagttttcgttccactgagcgtcagaccccgtagaaaagatcaaaggatcttcttgagatcctttttttctgcgcgtaatctgctgcttgcaaacaaaaaaaccaccgctaccagcggtggtttgtttgccggatcaagagctaccaactctttttccgaaggtaactggcttcagcagagcgcagataccaaatactgttcttctagtgtagccgtagttaggccaccacttcaagaactctgtagcaccgcctacatacctcgctctgctaatcctgttaccagtggctgctgccagtggcgataagtcgtgtcttaccgggttggactcaagacgatagttaccggataaggcgcagcggtcgggctgaacggggggttcgtgcacacagcccagcttggagcgaacgacctacaccgaactgagatacctacagcgtgagctatgagaaagcgccacgcttcccgaagggagaaaggcggacaggtatccggtaagcggcagggtcggaacaggagagcgcacgagggagcttccagggggaaacgcctggtatctttatagtcctgtcgggtttcgccacctctgacttgagcgtcgatttttgtgatgctcgtcaggggggcggagcctatggaaaaacgccagcaacgcggcctttttacggttcctggccttttgctggccttttgctcacatgttctttcctgcgttatcccctgattctgtggataaccgtattaccgcctttgagtgagctgataccgctcgccgcagccgaacgaccgagcgcagcgagtcagtgagcgaggaagcggaaga |
| pGGYL2 | gcgcccaatacgcaaaccgcctctccccgcgcgttggccgattcattaatgcagctggcacgacaggtttcccgactggaaagcgggcagtgagcgcaacgcaattaatgtgagttagctcactcattaggcaccccaggctttacactttatgcttccggctcgtatgttgtgtggaattgtgagcggataacaatttcacacaggaaacagctATTTAAATctagggagaccatgaccatgattacgccaagcttgcatgcctgcaggtcgactctagaggatccccgggtaccgagctcgaattcactggccgtcgttttacaacgtcgtgactgggaaaaccctggcgttacccaacttaatcgccttgcagcacatccccctttcgccagctggcgtaatagcgaagaggcccgcaccgatcgcccttcccaacagttgcgcagcctgaatggcgaatggcgcctgatgcggtattttctccttacgcatctgtgcggtatttcacaccgcatatggtgcactctcagtacaatctgctctgatgccgcatagGGTCTCaACAAATTTAAATttaagccagccccgacacccgccaacacccgctgacgcgccctgacgggcttgtctgctcccggcatccgcttacagacaagctgtgaccgtctccgggagctgcatgtgtcagaggttttcaccgtcatcaccgaaacgcgcgagacgaaagggcctcgtgatacgcctatttttataggttaatgtcatgataataatggtttcttagacgtcaggtggcacttttcggggaaatgtgcgcggaacccctatttgtttatttttctaaatacattcaaatatgtatccgctcatgagacaataaccctgataaatgcttcaataatattgaaaaaggaagagtatgagccatattcaacgggaaacgtcttgctctaggccgcgattaaattccaacatggatgctgatttatatgggtataaatgggctcgcgataatgtcgggcaatcaggtgcgacaatctatcgattgtatgggaagcccgatgcgccagagttgtttctgaaacatggcaaaggtagcgttgccaatgatgttacagatgagatggtcagactaaactggctgacggaatttatgcctcttccgaccatcaagcattttatccgtactcctgatgatgcatggttactcaccactgcgatccccgggaaaacagcattccaggtattagaagaatatcctgattcaggtgaaaatattgttgatgcgctggcagtgttcctgcgccggttgcattcgattcctgtttgtaattgtccttttaacagcgatcgcgtatttcgtctcgctcaggcgcaatcacgaatgaataacggtttggttgatgcgagtgattttgatgacgagcgtaatggctggcctgttgaacaagtctggaaagaaatgcataaacttttgccattctcaccggattcagtcgtcactcatggtgatttctcacttgataaccttatttttgacgaggggaaattaataggttgtattgatgttggacgagtcggaatcgcagaccgataccaggatcttgccatcctatggaactgcctcggtgagttttctccttcattacagaaacggctttttcaaaaatatggtattgataatcctgatatgaataaattgcagtttcatttgatgctcgatgagtttttctaactgtcagaccaagtttactcatatatactttagattgatttaaaacttcatttttaatttaaaaggatctaggtgaagatcctttttgataatctcatgaccaaaatcccttaacgtgagttttcgttccactgagcgtcagaccccgtagaaaagatcaaaggatcttcttgagatcctttttttctgcgcgtaatctgctgcttgcaaacaaaaaaaccaccgctaccagcggtggtttgtttgccggatcaagagctaccaactctttttccgaaggtaactggcttcagcagagcgcagataccaaatactgttcttctagtgtagccgtagttaggccaccacttcaagaactctgtagcaccgcctacatacctcgctctgctaatcctgttaccagtggctgctgccagtggcgataagtcgtgtcttaccgggttggactcaagacgatagttaccggataaggcgcagcggtcgggctgaacggggggttcgtgcacacagcccagcttggagcgaacgacctacaccgaactgagatacctacagcgtgagctatgagaaagcgccacgcttcccgaagggagaaaggcggacaggtatccggtaagcggcagggtcggaacaggagagcgcacgagggagcttccagggggaaacgcctggtatctttatagtcctgtcgggtttcgccacctctgacttgagcgtcgatttttgtgatgctcgtcaggggggcggagcctatggaaaaacgccagcaacgcggcctttttacggttcctggccttttgctggccttttgctcacatgttctttcctgcgttatcccctgattctgtggataaccgtattaccgcctttgagtgagctgataccgctcgccgcagccgaacgaccgagcgcagcgagtcagtgagcgaggaagcggaaga |
| pGGYL3 | gcgcccaatacgcaaaccgcctctccccgcgcgttggccgattcattaatgcagctggcacgacaggtttcccgactggaaagcgggcagtgagcgcaacgcaattaatgtgagttagctcactcattaggcaccccaggctttacactttatgcttccggctcgtatgttgtgtggaattgtgagcggataacaatttcacacaggaaacagctATTTAAATctagggagaccatgaccatgattacgccaagcttgcatgcctgcaggtcgactctagaggatccccgggtaccgagctcgaattcactggccgtcgttttacaacgtcgtgactgggaaaaccctggcgttacccaacttaatcgccttgcagcacatccccctttcgccagctggcgtaatagcgaagaggcccgcaccgatcgcccttcccaacagttgcgcagcctgaatggcgaatggcgcctgatgcggtattttctccttacgcatctgtgcggtatttcacaccgcatatggtgcactctcagtacaatctgctctgatgccgcatagGGTCTCaAGTTATTTAAATttaagccagccccgacacccgccaacacccgctgacgcgccctgacgggcttgtctgctcccggcatccgcttacagacaagctgtgaccgtctccgggagctgcatgtgtcagaggttttcaccgtcatcaccgaaacgcgcgagacgaaagggcctcgtgatacgcctatttttataggttaatgtcatgataataatggtttcttagacgtcaggtggcacttttcggggaaatgtgcgcggaacccctatttgtttatttttctaaatacattcaaatatgtatccgctcatgagacaataaccctgataaatgcttcaataatattgaaaaaggaagagtatgagccatattcaacgggaaacgtcttgctctaggccgcgattaaattccaacatggatgctgatttatatgggtataaatgggctcgcgataatgtcgggcaatcaggtgcgacaatctatcgattgtatgggaagcccgatgcgccagagttgtttctgaaacatggcaaaggtagcgttgccaatgatgttacagatgagatggtcagactaaactggctgacggaatttatgcctcttccgaccatcaagcattttatccgtactcctgatgatgcatggttactcaccactgcgatccccgggaaaacagcattccaggtattagaagaatatcctgattcaggtgaaaatattgttgatgcgctggcagtgttcctgcgccggttgcattcgattcctgtttgtaattgtccttttaacagcgatcgcgtatttcgtctcgctcaggcgcaatcacgaatgaataacggtttggttgatgcgagtgattttgatgacgagcgtaatggctggcctgttgaacaagtctggaaagaaatgcataaacttttgccattctcaccggattcagtcgtcactcatggtgatttctcacttgataaccttatttttgacgaggggaaattaataggttgtattgatgttggacgagtcggaatcgcagaccgataccaggatcttgccatcctatggaactgcctcggtgagttttctccttcattacagaaacggctttttcaaaaatatggtattgataatcctgatatgaataaattgcagtttcatttgatgctcgatgagtttttctaactgtcagaccaagtttactcatatatactttagattgatttaaaacttcatttttaatttaaaaggatctaggtgaagatcctttttgataatctcatgaccaaaatcccttaacgtgagttttcgttccactgagcgtcagaccccgtagaaaagatcaaaggatcttcttgagatcctttttttctgcgcgtaatctgctgcttgcaaacaaaaaaaccaccgctaccagcggtggtttgtttgccggatcaagagctaccaactctttttccgaaggtaactggcttcagcagagcgcagataccaaatactgttcttctagtgtagccgtagttaggccaccacttcaagaactctgtagcaccgcctacatacctcgctctgctaatcctgttaccagtggctgctgccagtggcgataagtcgtgtcttaccgggttggactcaagacgatagttaccggataaggcgcagcggtcgggctgaacggggggttcgtgcacacagcccagcttggagcgaacgacctacaccgaactgagatacctacagcgtgagctatgagaaagcgccacgcttcccgaagggagaaaggcggacaggtatccggtaagcggcagggtcggaacaggagagcgcacgagggagcttccagggggaaacgcctggtatctttatagtcctgtcgggtttcgccacctctgacttgagcgtcgatttttgtgatgctcgtcaggggggcggagcctatggaaaaacgccagcaacgcggcctttttacggttcctggccttttgctggccttttgctcacatgttctttcctgcgttatcccctgattctgtggataaccgtattaccgcctttgagtgagctgataccgctcgccgcagccgaacgaccgagcgcagcgagtcagtgagcgaggaagcggaaga |

**Figure S1**. The stability investigation of the selected strains used for the next round of transformation including LYW1-11, LYW2-11, LYW3-5, LYW4-2, LYW5-2, LYW6-3 and the final optimized strain LYW7-3.
